# Chrysoeriol Chemosensitized Renal Cell Carcinoma (RCC) Cell Lines to Trigger TRAIL Induced Apoptosis In Vitro by Up‐Regulating Pro‐Apoptotic and Down‐Regulating Anti‐Apoptotic Genetic Factors

**DOI:** 10.1002/fsn3.70442

**Published:** 2025-07-16

**Authors:** Zahra Batool, Qihang Wu, Mohammad Amjad Kamal, Guobin Weng, Bairong Shen

**Affiliations:** ^1^ Department of Urology and Institutes for Systems Genetics, Frontiers Science Center for Disease‐Related Molecular Network, West China Hospital Sichuan University Chengdu China; ^2^ Department of Food Science Huazhong Agriculture University Wuhan China; ^3^ Department of Urology Ningbo Yinzhou No. 2 Hospital Ningbo Zhejiang China

**Keywords:** FOXO1, mRNA expression, oxidative stress, protein expression, renal cancer, renal carcinoma, TRAIL

## Abstract

Renal carcinoma is a lethal cancer, researched by several studies to get insights into the molecular causes of disease, in order to come up with advanced therapeutic treatments. Tumor necrosis factor‐related apoptosis‐inducing ligand (TRAIL), is an anticancer cytokine posing therapeutic effects to treat cancer. However, certain cancer types including renal cell carcinoma developed resistance towards TRAIL, hence limiting its usefulness in cancer treatment. Recently, synergistic approach has been emerged in clinical settings to sensitize cancer cells towards TRAIL treatment by combining with natural antioxidants and anti‐inflammatory compounds for providing chemo‐sensitizing effects. Hence, this study has used chemo‐sensitizing effect of chrysoeriol for the very first time to sensitize renal carcinoma cell lines for treatment with TRAIL by synergistic treatment approach, in vitro. The effects of 20 μM chrysoeriol, 50 ng/mL TRIAL and their synergistic combination were investigated deeply by adopting different experimental strategies in vitro. Synergistic combination of 20 μM chrysoeriol/50 ng/mL TRIAL provided apoptosis of TRAIL resistant cell lines which was confirmed by increasing the expressions of caspases including caspase‐3 and caspase‐8 and caspase‐9, increasing the expression of interleukins 10, while decreasing the expression of interleukins 6, triggering cellular apoptosis, inhibiting proteasome activity, lossing mitochondrial membrane potential and triggering cytchrome C release. Meanwhile, rise in death receptor 4 expression as well as up‐regulation of pro‐apoptotic and down‐regulation of anti‐apoptotic genes further provided evidence for chemo‐sensitizing effect of chrysoeriol on TRAIL resistant renal carcinoma cells to trigger apoptosis. Hence, chrysoeriol could be employed in the anticancer drug development for therapeutic treatment of renal cancer.

## Graphical abstract

Recently, a synergistic approach has emerged in clinical settings to sensitize cancer cells towards TRAIL treatment by combining it with some natural antioxidants and anti‐inflammatory compounds for providing chemo‐sensitizing effects. Hence, this study has used the chemo‐sensitizing effect of chrysoeriol for the very first time to sensitize renal carcinoma cell lines for treatment with TRAIL by a synergistic treatment approach, in vitro.

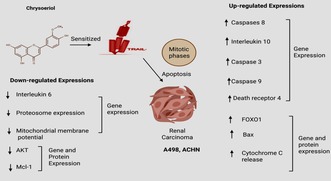

**Abbreviations**
786‐O: human renal cell carcinoma cell line 786‐O
AKT: protein kinase B
AMC: 7‐amino‐4‐methyl‐coumarin
Apaf‐1: apoptotic protease activating factor‐1
Bax: Bcl‐2‐associated X protein
Bcl‐2: B‐cell lymphoma 2
Caki‐1: clear cell renal carcinoma cell line 1
CCK‐8: cell counting Kit‐8
cellular FLICE: (FADD‐like IL‐1β‐converting enzyme)‐inhibitory protein (cFLIP)
chrysoeriol: 3′‐O‐methoxy flavone polyphenol
CI: combination index
CKI: cyclin‐dependent kinase inhibitor
Ct: cycle threshold
DEVDase: Asp‐Glu‐Val‐Asp‐ase
DISC: death inducing signaling cascade
DR: death receptors
FADD: Fas associated death domain
FBS: fetal bovine serum
FOXO1: forkhead box O1
G1: growth phase
G2: phase (cell replenishing phase for synthesizing protein for chromatin manipulation phase)
HRP: horseradish peroxidase
IAPs: **i**nhibitor of apoptosis proteins
ILs: Interleukin's
MAPK: mitogen‐activated protein kinase
MCL‐1: myeloid cell leukemia 1
MEM: minimal essential medium
MMP: mitochondrial membrane potential
MOMP: mitochondrial outer membrane permeabilization
NF‐κB: nuclear factor‐kappa B
PAGE: polyacrylamide gel electrophoresis
PI: propidium iodide
qRT‐PCR: quantitative reverse transcription polymerase chain reaction
RCC: renal cell carcinoma
RCC4: renal cell carcinoma cell line 4
ROS: reactive oxygen species
S phase: DNA synthesis phase
SUC‐LLVY‐AMC: N‐succinyl‐Leu‐Leu‐Val‐tyr‐amino‐4‐methyl‐coumarin
TMB: tetra, methylbenzidine
TRAIL: Tumor necrosis factor‐related apoptosis‐inducing ligand
ΔCt: delta Ct

## 1 Introduction

Renal cell carcinoma (RCC) is a lethal type of renal cancer responsible for approximately 90 to 95% of deaths worldwide (Obaidi et al. 2020). An advanced stage of RCC carries a poor prognosis due to complex clinical manifestations as well as resistance towards chemotherapies, leading to only a 5% overall survival rate in five years in patients (Chae et al. 2020; Padala et al. 2020). Renal cancer treatments often aim to modulate the expression of specific interleukins, cytokines, and genes that can trigger apoptosis for eliminating cancer cells (Murata et al. 2012; Klaunig et al. 2010). This process involves multiple signaling pathways where cancer cells disrupt their own responses to environmental signals, such as growth factors or immune responses, leading to uncontrolled proliferation. Anticancer drug therapies exploit this mechanism by using pharmaceutical agents that either enhance pro‐apoptotic signals or inhibit survival signals within cancer cells (Huo et al. 2014; Korbecki et al. 2013). However, optimizing these treatments to selectively enhance apoptosis in cancer cells while minimizing damage to healthy tissues remains a significant challenge, highlighting the need for targeted therapeutic strategies (Matsuzawa and Ichijo 2008). These targeted therapies can selectively target cancer cells without affecting healthy cells by using combination therapies that target multiple pathways simultaneously, personalizing treatment based on a patient's genetic profile, or developing novel drug delivery systems. Hence, research should focus on enhancing the specificity of these therapies, developing combination strategies that engage multiple pathways, and leveraging personalized medicine to tailor treatments based on individual patient profiles (Chae et al. 2020).

**FIGURE 1:**
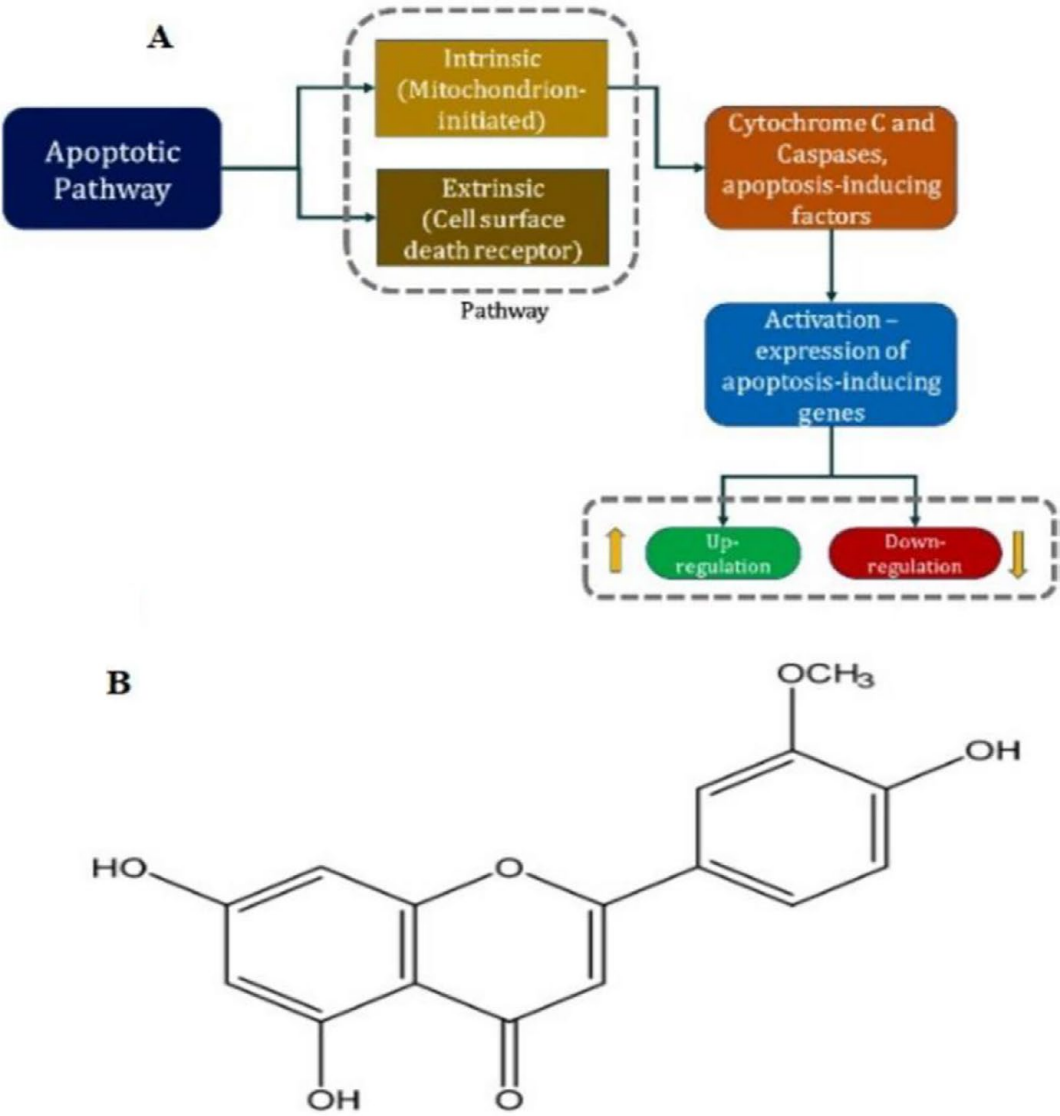
Apoptotic pathways and chrysoeriol structural conformation. (A) Intrinsic and extrinsic pathways of apoptosis. (B) Chrysoeriol structural conformation as a polyphenolic flavone. Apoptotic pathways and chrysoeriol structural conformation. (A) Intrinsic and extrinsic pathways of apoptosis. (B) Chrysoeriol structural conformation as a polyphenolic flavone.

Moreover, apoptosis occurred through two primary pathways: an intrinsic pathway, originating within the cell, and an extrinsic pathway, initiated by external signals. The intrinsic pathway, primarily regulated by mitochondria, is activated by various internal stress signals. In contrast, the extrinsic pathway is triggered by external factors interacting with cell surface death receptors (Matsuzawa and Ichijo 2008; Batool et al. 2021; Jeon 2017) (Figure 1A). During intrinsic apoptosis, cytochrome C and specific proteases are released from the mitochondrial intermembrane space into the cytoplasm, initiating the apoptotic cascade. Additionally, various factors released from the mitochondria modulate receptor activity, ultimately influencing the expression of apoptosis‐inducing genes (Micucci et al. 2015).

Additionally, certain targeted therapies are being introduced into clinical practice due to their ability to selectively target cancer cells and induce apoptosis. Tumor necrosis factor‐related apoptosis‐inducing ligand (TRAIL) is one such targeted therapy, known for its ability to inhibit various tumor types while causing minimal toxicity to normal human cells (Hassanzadeh et al. 2018). TRAIL, functioning as a trimeric ligand, binds to death receptors DR4 (TRAIL‐R1) and/or DR5 (TRAIL‐R2), triggering the death‐inducing signaling complex (DISC) and recruiting Fas‐associated death domain (FADD). FADD subsequently activated initiator procaspase‐8, leading to the activation of caspase‐8. This activation amplified the death signal by cleaving and activating other caspases, including procaspase‐3. Active caspase‐3, known as an executioner caspase, degraded cellular cytoplasmic and nuclear proteins, driving apoptosis (Dimberg et al. 2013). In spite of the great ability of TRAIL to induce apoptosis in cancer cells, certain cancer types, particularly renal cancer, are developing resistance due to several factors. One primary reason is the downregulation or mutation of death receptors (DR4 and DR5) on the cell surface, which reduced TRAIL's ability to initiate the apoptotic signaling pathway. Additionally, overexpression of anti‐apoptotic proteins such as cellular FLICE (FADD‐like IL‐1β‐converting enzyme)‐inhibitory protein (c‐FLIP), B‐cell lymphoma 2 (Bcl‐2), and inhibitor of apoptosis proteins (IAPs) inhibited caspase activation, preventing apoptosis. Moreover, defects in the mitochondrial apoptotic pathway, including impaired cytochrome C release and reduced mitochondrial membrane potential, further contributed to resistance. The imbalance between pro‐apoptotic and anti‐apoptotic signaling pathways, along with enhanced survival signaling through nuclear factor‐kappa B (NF‐κB), protein kinase B (AKT), and mitogen‐activated protein kinase (MAPK) pathways, also supported the survival of TRAIL‐resistant renal carcinoma cells (Lemke et al. 2014).

However, TRAIL resistance has been shown to decrease when used in combination with other drugs, such as bortezomib and proteasome inhibitors. Although bortezomib enhances TRAIL's apoptotic effects, its high toxicity limits its clinical use, highlighting the need for alternative synergistic partners. These alternatives should possess TRAIL‐sensitizing activity while maintaining relatively lower toxicity (Henrich et al. 2015). Subsequently, natural antioxidants such as curcumin have also been reported to sensitize renal cancer cells to TRAIL‐induced apoptosis mainly via mediation of antioxidative mechanisms (Obaidi et al. 2020), and thymoquinone, being another natural antioxidant, directly induced apoptosis in renal carcinoma cells (Chae et al. 2020). However, these studies mainly focused on targeting antioxidative mechanisms in the cancer cells; hence, further research is warranted on a broader scale on the effect of natural antioxidants on TRAIL‐induced apoptosis by affecting multiple cellular mechanisms.

Chrysoeriol (3’‐O‐methoxy flavone polyphenol) is a naturally derived flavonoid belonging to the flavone chemical superfamily (Figure 1B). It is structurally derived from luteolin and has been extensively studied for its diverse pharmacological activities, including anti‐inflammatory and anti‐proliferative effects (Min et al. 2020; Aboulaghras et al. 2022). Globally, its anticancer properties have drawn significant attention, with studies highlighting its ability to induce apoptosis in various cancers, including lung, cervical, and colon cancers (Wei et al. 2019; Zeng et al. 2016). This global relevance is attributed to the high prevalence of these cancers worldwide, making chrysoeriol a promising candidate for anticancer therapy. Chrysoeriol's anticancer effects are primarily due to its strong antioxidant capabilities, enabling it to neutralize free radicals, chelate metal ions, and inhibit enzymes responsible for generating reactive oxygen species (ROS).

However, despite its promising therapeutic potential, chrysoeriol is not without limitations. It can pose potential side effects, including gastrointestinal disturbances, allergic reactions, and cytotoxicity at higher doses. Hence, emphasizing the importance of optimizing its dosage and formulation for safe and effective use. Given the rising global burden of cancer, particularly in low‐ and middle‐income countries, the exploration of naturally derived compounds like chrysoeriol is of immense significance for developing accessible and affordable anticancer therapies.

Interestingly, TRAIL‐resistant RCC cell lines, including A498 and ACHN, are critical models for investigating therapeutic strategies against resistant cancer types. These two cell lines are widely recognized for their resistance to TRAIL‐induced apoptosis, making them ideal for exploring mechanisms of resistance and testing potential treatments. Other RCC cell lines, such as clear cell renal carcinoma cell line 1 (Caki‐1), human renal cell carcinoma cell line 786‐O (786‐O), and renal cell carcinoma cell line 4 (RCC4), also exhibited TRAIL resistance due to various mechanisms, including overexpression of anti‐apoptotic proteins (Caki‐1), mutations in apoptotic pathways (786‐O), or hypoxia‐related resistance (RCC4). However, A498 and ACHN are particularly significant because they not only exhibited TRAIL resistance but also represented clear models for studying molecular dynamics of apoptosis resistance in RCC. Understanding the response of these cell lines to TRAIL, chrysoeriol, and their synergistic combination could provide critical insights into overcoming resistance in RCC, paving the way for the development of more effective therapeutic strategies. Therefore, TRAIL‐resistant cell lines A498 and ACHN were chosen to investigate the effects of TRAIL, chrysoeriol, and their synergistic combination for the very first time on cellular morphology, apoptosis, caspase expression, cytokine expression, proteasome activity, mitochondrial membrane potential, and cytochrome C release in vitro. Meanwhile, down‐regulation and up‐regulation of specific genes for triggering apoptosis of RCC cells were also investigated deeply in vitro.

Moreover, the novelty of the research lies in demonstrating the potential of chrysoeriol as a sensitizer for TRAIL in overcoming resistance in TRAIL‐resistant RCC cell lines, A498 and ACHN. The research uniquely established that chrysoeriol significantly enhanced TRAIL's apoptotic effects on resistant cancer cells. Unlike previous studies that tested various sensitizers without fully understanding their mechanisms, this study provided a detailed, multi dimensional analysis of how chrysoeriol exerted its sensitizing effects by investigating its synergistic effects on different time durations which provided comprehensive results of synergistic effects both TRAIL and chrysoeriol. Interestingly, chrysoeriol exerted its sensitizing effects by strong antioxidant capabilities, enabling it to neutralize free radicals, chelate metal ions, and inhibited enzymes responsible for ROS. Meanwhile, study's novelty is further highlighted by its extensive exploration of multiple apoptosis‐related parameters, making it one of the most comprehensive investigations in this context. It simultaneously examined cellular morphology, apoptosis induction, caspase expression, cytokine expression, proteasome activity, mitochondrial membrane potential, and cytochrome C release, allowing for a complete understanding of the apoptotic process over different time durations, hence providing robust evidence of the compound's effectiveness and revealing the interconnected pathways through which it promotes cell death.

## 2 Materials and Methods

The study included protocol design for in vitro experiments, following cell viability, synergy analysis, cells morphology, apoptosis determination by flow cytometry, ELISA for cytokines, Asp‐Glu‐Val‐Asp‐ase (DEVDase) assays for caspase‐3 and caspase‐9 expression using respective ELISA kits, assessment of proteasome activity by fluorometric assay, assessment of mitochondrial membrane potential assays using flow cytometry, cytochrome C release using western blot analysis, RNA extraction, and quantitative reverse transcription polymerase chain reaction (qRT‐PCR) for specific genes expression and western blotting for determining protein expression of specific genes after synergistic treatments of renal carcinoma cells. The details of all experiments along with experimental chemicals and used apparatus are discussed extensively in this section.

### 2.1 Culturing of Cell Lines

Both cell lines Clear cell (A498) and Papillary renal cell carcinoma (ACHN) cell lines were bought from Pro‐cell company (Hubei, China) and cultured in 25 cm^2^ T flasks in their specific recommended culture medium formulated with 90% minimal essential medium (MEM) for both A498 and ACHN cell lines. A 10% fetal bovine serum (FBS) together with a 100 units/mL penicillin and a 100 units/mL streptomycin obtained from Sigma Aldrich (St. Louis, USA) were used to augment both media. After culturing, a humidified incubator with 5% CO_2_ (HeracellTM 150i, ThermoFisher Scientific) was used to incubate cells. After 24 h of culturing, cell growth was carefully observed to get them in the logarithmic growth phase of 10–30 passages for further experiments.

### 2.2 Preparation of Treatment Solutions

A 50 mM stock solution of chrysoeriol (Sigma‐Aldrich, St. Louis, MO, USA) was prepared by dissolving 50 mg of chrysoeriol powder in 1 mL of DMSO (Sigma‐Aldrich, St. Louis, MO, USA), which resulted in a final concentration of 50 mM. This stock solution was then diluted in culture medium at a 1:2000 (v/v) ratio, achieving a final DMSO concentration of approximately 0.05%, which served as a negative control. The selection of chrysoeriol concentration was based on preliminary dose–response experiments, where a range of concentrations (0.01–1 mM) was tested on cell lines Clear cell (A498) and Papillary renal cell carcinoma (ACHN) cell lines. Results indicated that 0.5 mM chrysoeriol significantly enhanced apoptotic sensitivity without causing notable cytotoxicity to normal cells, making it the optimal concentration for further experiments.

Similarly, Sorafenib (Sigma‐Aldrich, St. Louis, MO, USA) was prepared at a final concentration of 10 mM, which was selected as a positive control due to its well‐established efficacy in inducing apoptosis in renal carcinoma cells. The concentration of TRAIL (500 μg/mL) was determined through a series of optimization experiments. Specifically, TRAIL concentrations ranging from 50 to 1000 ng/mL were evaluated for their ability to induce apoptosis in A498 and ACHN cells. A concentration of 500 ng/mL was found to induce a significant apoptotic response in these TRAIL‐resistant cells without causing excessive non‐specific toxicity, making it the most suitable for this study by adopting and modifying the previously reported study (Obaidi et al. 2020). Then final working concentrations for chrysoeriol and TRAIL were prepared in the respective culture medium of the A498 and ACHN cell lines.

### 2.3 Performing Assay to Determine Cells Viability

Cell viability was assessed using cell counting kit‐8 (CCK‐8, Biosharp Life Sciences) due to its high sensitivity, simplicity, and non‐toxic nature, which allows continuous monitoring of cell viability without interfering with cellular processes. Unlike other viability assays (such as MTT or trypan blue exclusion), CCK‐8 offered superior sensitivity due to its water‐soluble tetrazolium salt, which produced a highly stable, water‐soluble formazan dye upon reduction by cellular dehydrogenases. This minimized assay variability and allowed for direct absorbance measurement without requiring solubilization steps.

A498 and ACHN were seeded at a density of 7.5 × 10^5^ cells/mL in 96‐well plates and allowed to adhere for 24 h. After this incubation period, the cells were treated with culture medium containing DMSO (negative control) or different concentrations of chrysoeriol (10, 15, and 20 μM) for 5 h. Subsequently, the cells were exposed to TRAIL at concentrations of 0, 10, 25, 50, and 75 ng/mL for an additional 19 h, making a total treatment duration of 24 h.

Following the treatment period, 10 μL of the CCK‐8 reagent was added to each well, and the plates were incubated at 37°C for 4–5 h under standard conditions. Absorbance was then measured at 450 nm using a SpectraMax M2 plate reader (Molecular Devices LLC, USA). Cell viability was calculated as the percentage of the control (DMSO‐treated cells), using theformula:

$$\text{Cell Viability}\;\left(\%\right)=\text{Absorbance of Treated Cells}/\text{Absorbance of Control Cells}\times 100$$

Moreover, statistical analysis was performed using Origin 8.5 Software. Data were presented as mean ± standard deviation (SD) of three independent experiments. Comparisons between groups were conducted using one‐way analysis of variance (ANOVA) followed by Tukey's post hoc test. A *p*‐value < 0.05 was considered statistically significant.

### 2.4 Evaluation of Treatments Synergistic Effects by Combination Index Analysis

The synergistic anti‐cancer activity of chrysoeriol and TRAIL combination was determined by combination index (CI) analysis based on median effect. CI of interaction types between chrysoeriol and TRAIL was calculated by CalcuSyn (Biosoft, Ferguson, MO, USA) software. CI = 1 indicated an additive effect, CI > I indicated antagonism; however, CI < 1 indicated a synergistic interaction. In addition to this, the degree of synergy was mainly considered by referring to the previous study of (Chou and Talalay 1984) to determine drugs synergistic effects, which indicated +++ as strong synergy, ++ as medium, and + as low synergy.

### 2.5 Cells Morphology

Cell lines were seeded and cultured in a 25 cm^2^ tissue culture flasks supplemented with their specified culture medium along with 10% FBS. Cell culture plates were placed in a humidified incubator for 24 h, followed by washing thrice with PBS. 1 mL of 0.25% Trypsin–EDTA (Sigma Aldrich) was used to detach cells from the culture plates for about 4 to 5 min. Then cell pellets were suspended by centrifuging the cell solution in 15 mL sterile polyethylene tubes for 4 min. Afterwards, cells were seeded in the 6‐well plates with their added growth culture medium at a density of 1 × 10^6^ cells/well. After 24 h of culture, 20 μM of chrysoeriol, 50 ng/mL TRAIL, and their best synergistic combination of 20 μM chrysoeriol/50 ng/mL TRAIL were added to the 6‐well plates, separately, containing the cultured cell lines (A498, ACHN). After 24 h of incubation in a humidified (5% CO_2_) incubator at 37°C, the entire 6‐well plates were taken out, followed by observation of every cell type with a fluorescence microscope (Model: Olympus‐IX71, Japan). Obtained micrographs were saved in a picture files folder.

### 2.6 Assays to Determine Cellular Apoptosis

Annexin V‐FITC/propidium iodide dyes were used for detection and quantification of cellular events associated with apoptosis on both cell lines, followed by cell sorting with a flow cytometer (Biopharmaceutical Technology Co. Ltd., China and FACSCalibur, Becton Dickson, San Jose). Cells were cultured in 6‐well plates, at a density of 1 × 10^6^ cells per well, followed by keeping them in a humidified incubator overnight. After that, cultured cells were treated with 0.05% DMSO as a control, 20 μM of chrysoeriol, 50 ng/mL TRAIL, and their best synergistic combination of 20 μM chrysoeriol/50 ng/mL TRAIL and kept in incubation for 24 h. After 24 h, cells were removed from the incubator and dissociated with trypsin, followed by washing them with normal saline solution and centrifugation at 1200 × g for 5 min to separate the supernatant from 6‐well plates. Afterwards, cells were suspended with 500 μL of binding buffer for 5 min at 4°C, followed by the addition of 5 μL of annexin V‐FITC. It was further proceeded by placing the plates in the dark for storage for 10 min, followed by the addition of 10 μL of propidium iodide (PI) dye and subsequent incubation at 20°C. A flow cytometer was used to determine programmed cell death once cells were sorted. Then, FACSDiva software's FITC (green) emission recorder 515–545 nm and PI (red) emission recorder of 600 nm were used for measuring the apoptosis. Approximately, 1000 events were recorded for every measurement. Obtained results, including all data, were then analyzed using flowjo version 10.40 for their graphical representation.

### 2.7 Performing ELISA to Determine Cytokines

Post combination activities of interleukin‐6 (IL‐6) and interleukin‐10 (IL‐10) were determined with corresponding ELISA kit (mlbio Shanghai Enzyme‐linked Biotechnology Company). Before performing assay, A498 and ACHN were seeded in six well plates and treated with 20 μM/50 ng/mL of chrysoeriol/TRAIL to investigate post combination effect after 3, 7, 12, and 24 h of treatment. Cells were detached using trypsin–EDTA from a 25 cm^2^ T‐flask after different time intervals (3, 7, 12, and 24) and moved to 1.5 mL centrifuge tube to perform ELISA. Cytokines ELISA was performed in accordance with directions given by the ELISA kits manufacturers. All quantification was performed by standard curve generation by microplate reader, based on their measurement of OD values, directly proportional to their expression levels.

### 2.8 Determination of Asp‐Glu‐Val‐Asp‐Ase (DEVDase) Activity by DEVDase Assay

Post combination activities of caspase‐3 and caspase‐9 were determined with corresponding ELISA kit (BioVision Inc. Milpitas, California, USA). Before performing assay, A498 and ACHN were seeded in six well plates and treated with 20 μM/50 ng/mL of chrysoeriol/TRAIL to investigate post combination effect after 3, 7, 12, and 24 h of treatment. Cells were detached using trypsin–EDTA from 25 cm^2^ T‐flask after different time intervals (3, 7, 12, and 24) and moved to 1.5 mL centrifuge tube to perform ELISA. Furthermore, cells washing was performed with different saline solutions followed by centrifugation at 1500 × g (SorvallTM legendtm micro 21R, Thermo Fisher Scientific) for 5 min for removing supernatant. In addition to this, further added cells extraction buffer (1 mL) in cell lines culture medium and incubated chilled using ice for half an hour. At 4°C, centrifugation of cells was carried out at 13,000 × g for 10 min. Six varying concentrations of 100 μL standards, controls and cells that underwent lysis were placed into 96 well plate and taken through incubation for 2 h at 20°C. Then wells were washed using IX washing buffer containing 0.01 M phosphate‐buffered saline and 0.05% polysorbate 20. After that, caspase‐3 (100 μL) and caspase‐9 (100 μL) antibodies solution was added to the 96‐well plates and incubated for 1 h at normal room temperature. Afterwards, wells were washed by using 1X washing buffer solution in quantuplicate. 1X rabbit IG horseradish peroxidase (HRP) (100UL) was later added and subsequently incubated the wells for 1 h at room temperature. 100 μL of tetramethylbenzidine (TMB) substrate solution was poured into each well and allowed to incubate for half an hour in the dark at 20°C. The reaction in each well was stopped by adding 100 μL of 1 N sulfuric acid and subsequently absorbance was determined at 450 nm using micro‐plate reader. Moreover, quantification was performed after constructing standard curve for caspase‐3 and caspase‐9 in accordance with the OD measurement.

### 2.9 Proteasome Assay

A498 and ACHN cell lines were cultured in 6‐well plates at a density of 1 × 10^6^ cells per well, followed by keeping them in a humidified (5% CO_2_) incubator overnight. After that, cultured cells were treated with 0.05% DMSO as a control, 20 μM of chrysoeriol, 50 ng/mL TRAIL, and their best synergistic combination of 20 μM chrysoeriol/50 ng/mL TRAIL and kept in incubation for 24 h. After 24 h of culturing, cells were lysed in proteasome lysis buffer supplied by Sigma Aldrich, St. Louis, MO, USA with a composition of 10 mM NaCl, 50 mM HEPES pH 7.8, 0.2% Triton‐X100, I mM EDTA, 1.5 mM MgCl_2_, 250 mM sucrose, and DTT 5 mM. All lysates were transferred into ice pre‐chilled Eppendorf tubes, followed by sonication for 10 s using a microtip set on ~2. Afterwards, centrifugation was performed at 10,000 × g for 10 min at 4°C, followed by incubation of the supernatant with 100 μM N‐succinyl‐Leu‐Leu‐Val‐tyr‐amino‐4‐methyl‐coumarin (SUC‐LLVY‐AMC) fluorogenic substrate in the proteasome lysis buffer. Moreover, ATP solution obtained from Sigma Aldrich, St. Louis, MO, USA was added to the proteasome lysis buffer in order to obtain a final concentration of 2 mM before substrate addition. Afterwards, 7‐amino‐4‐methyl‐coumarin (AMC) was measured fluorometrically at an excitation wavelength of 360 nm and an emission wavelength of 475 nm. The assay was measured kinetically for 60 cycles per minute for 60 min at 37°C. Meanwhile, 100 mM MG‐122 was added to the 0.05% DMSO treated cell lysates, serving as a positive control, in order to ensure the proteasome‐derived activity. Afterwards, normalization of results was done by performing a BCA protein assay (Sigma Aldrich, St. Louis, MO, USA).

### 2.10 Assessment of Mitochondrial Membrane Potential

Flow cytometry was used to determine the mitochondrial membrane potential in differently treated cells. A498 and ACHN cell lines treated by 0.05% DMSO treated controls and 20 μM chrysoeriol/50 ng/mL TRIAL were taken through a 5‐min incubation using rhodamine 123 (mlbio Shanghai Enzyme‐linked Biotechnology Co. Ltd.) at 37°C under dark conditions. Rhodamine 123 was absorbed by cells mitochondria in proportion to their membrane potential and subsequently harvested in a suspension of phosphate buffer solution before the eventual use of flow cytometry.

### 2.11 Cytochrome C Release Determination

Ice cold phosphate buffer solution was used to wash the cultured cells (0.05% DMSO treated controls, and 20 μM chrysoeriol/50 ng/mL) TRIAL and then dissolved in the lysis buffer (80 mL) [10 mM KCl, 1 mM DTT, 250 mM Sucrose, 1 mM EDTA, 20 mM triphosphate hydrochloric acid (pH 7.2), 10 mg/mL leucinease, 1.5 mM MgCl_2_, 2 mg/mL aprotinin for 2 min and 5 mg/mL leucinease]. A 10 min centrifugation was carried out on the lysates at 12,000 g to separate the cell pellets and supernatant. The obtained cytosolic fractions were further used for western blot with an anti‐cytochrome c antibody. The detail of the western blot protocol is described in the specific western blot section below.

### 2.12 Extraction of RNA

A498 and ACHN cell lines were treated with 0.05% DMSO as a control, 20 μM of chrysoeriol, 50 ng/mL TRAIL, and their best synergistic combination of 20 μM chrysoeriol/50 ng/mL TRAIL to extract RNA for determining the effect of these treatments to evaluate the expression of death receptor 4 (DR4). However, only 0.05% DMSO treated controls and 20 μM chrysoeriol/50 ng/mL TRAIL treated cells were used to extract RNA from cells for expression analysis of AKT1, FOXO1, Mcl‐1, and Bax. RNA extraction was performed using E.Z.N.A. Total RNA Kit 1 (Omega biotech, USA) in accordance with the instructions in the kit manual. Additionally, all reagents used for performing real‐time PCR and making cDNA from mRNA were purchased from the ClonTech Takara Cellartis (China).

### 2.13 Quantitative Reverse Transcription Polymerase Chain Reaction (qRT‐PCR)

Selected genes expression of different genes including Homosapeins AKT1 with Forward: 5′ ATGGAGAACACTGAAAACTC‐3′, Reverse: 5′‐ATACTGTTTCAGCATGGCAC‐3′, Homosapeins FOXO1 with Forward: 5′‐ATGGACGAAGCGGATCAACG‐3′, Reverse: 5′‐TTATGATGTTTTAAAGGGAA‐3′, Homosapeins MCL‐1 with Forward: 5′‐ATGTTTGGCCTCAAAAGAGA‐3 and Reverse: 5′‐TTACAGTAAGGCTATCAAAT‐3′, Homosapeins Bax with Forward: 5′‐ATGGACGGGTCCGGGGAGCA‐3′ and Reverse: 5′‐TCAGCCCATCTTCTTCCAGA‐3′, Homosapeins DR4 with Forward: 5′‐ATGGACGCACTGATCAACTC‐3′ and Reverse: 5′‐ATAATCAAGCATTCGCAGAC‐3′ and Homosapeins Beta actin with Forward: 5′‐TTCCAGCCTTCCTTCCTGGG‐3′ and Reverse: 5′‐AGGAGCAATGATCTTGATCT‐3′ primers were obtained through a qRT‐PCR using LightCycler 480 system. Primer Premier 5 Software was used to design the primer sequences of respective genes. After designing the sequences were sent to the Shangong Biotechnology Co. Ltd. for making the primer.

Primer validation was performed prior to experimental use to ensure their specificity, efficiency, and reliability. Each primer pair was initially validated using melt curve analysis, which confirmed the production of a single, sharp peak for each gene, indicating high specificity without any non‐specific amplification products. The efficiency of each primer pair was determined by generating standard curves using serial dilutions of cDNA (5‐point dilution series), with efficiency values ranging between 95% and 105%, meeting the recommended range of 90%–110% for reliable qRT‐PCR analysis. The correlation coefficient (*R* ^2^) of each standard curve exceeded 0.98, demonstrating excellent linearity across the dilution range. Furthermore, amplification curves were carefully examined, confirming the absence of primer‐dimer formation. Following validation, qRT‐PCR was conducted, and cycle threshold (Ct) values for each gene were determined. Beta‐actin was used as an internal reference gene, and relative gene expression was calculated using the delta Ct (ΔCt) method, providing a quantitative measure of gene expression as relative fold change compared to the reference gene.

### 2.14 Performing Western Blot to Determine Protein Expression

Cultured A498 and ACHN cell lines were treated with 0.05% DMSO as a control and 20 μM chrysoeriol/50 ng/mL TRIAL and then kept for 24 h in a humidified incubator. After 24 h, the cells were washed with ice‐cold PBS and lysed on ice in 50 mL of lysis buffer (50 mM Tris–HCl, 1 mM EGTA, 1% Triton X‐100, 1 mM phenylmethylsulfonyl fluoride, and pH 7.5). Regarding the western blot of cytochrome c release, the pre‐treatment of cells is described in the above section (cytochrome c release). However, the remaining western blot protocol in the following was the same for all proteins, and an explanation is given in the following.

Cells lysates were centrifuged at 10,000 g for 15 min at 4°C, and supernatant fractions were collected. Proteins were separated by 10%–12% SDS‐polyacrylamide gel electrophoresis (PAGE) and transferred to an Immobilon‐P membrane, followed by incubation with a specific antibody. Specific proteins were detected using an enhanced chemiluminescence (ECL) western blot kit (EMD Millipore, Germany) according to the manufacturer's instructions, and visualization was performed with Imagequant LAS 4000 (Life Science, Uppsula, Sweden). Meanwhile, loading differences were normalized by using anti‐β‐actin antibody. Greyscaling and densitometry analysis was done using ImageJ 1.50i software (National institute of health).

Moreover, antibodies used in this study were carefully validated for specificity and reliability. For each target protein, a distinct band at the expected molecular weight was observed. The antibody for *Homo sapiens* AKT1 showed a clear band at approximately 60 kDa, Forkhead Box O1 (FOXO1) at 70 kDa, Myeloid Cell Leukemia 1 (MCL‐1) at 37 kDa, Bcl‐2‐associated X protein (Bax) at 21 kDa, DR4 at 55 kDa, and cytochrome C at 15 kDa in mitochondrial fractions. β‐actin, used as the loading control, was detected at 42 kDa. Antibodies were initially validated using positive control lysates known to express the respective target proteins, ensuring no non‐specific binding. Secondary antibody validation was carried out by performing control blots in which the primary antibody was omitted, showing no bands and confirming the specificity of secondary antibody binding. Optimization of antibody dilutions and blocking agents was performed to achieve the best signal‐to‐noise ratio, with final dilutions selected based on clear, strong target bands and minimal background. Additionally, the validation process included melt curve analysis and optimization of blocking agents to minimize background. All western blot experiments were conducted with consistent protein loading, as confirmed by uniform β‐actin bands across all samples. The resulting protein expression levels were quantified and normalized to β‐actin, and statistical analysis of the data was performed using densitometry.

### 2.15 Statistically Analyzed Data by Different Software's

Data obtained from different studies was represented as mean (±) standard deviation of three replications each time. These SD were calculated by using Microsoft Excel version 2017. The significant differences (*p* < 0.05, *p* < 0.01, and *p* < 0.001) were calculated by one‐way and two‐way ANOVA from SPSS7 version 7 Software, where a probability of 0.05 or less was found statistically significant. Meanwhile, the designing of all graphs was done by using Origin 8.5 Software.

## 3 Results and Discussion

### 3.1 Determined Cytotoxicity of Renal Cancer Cell Lines by Combination Index Synergy

**FIGURE 2:**
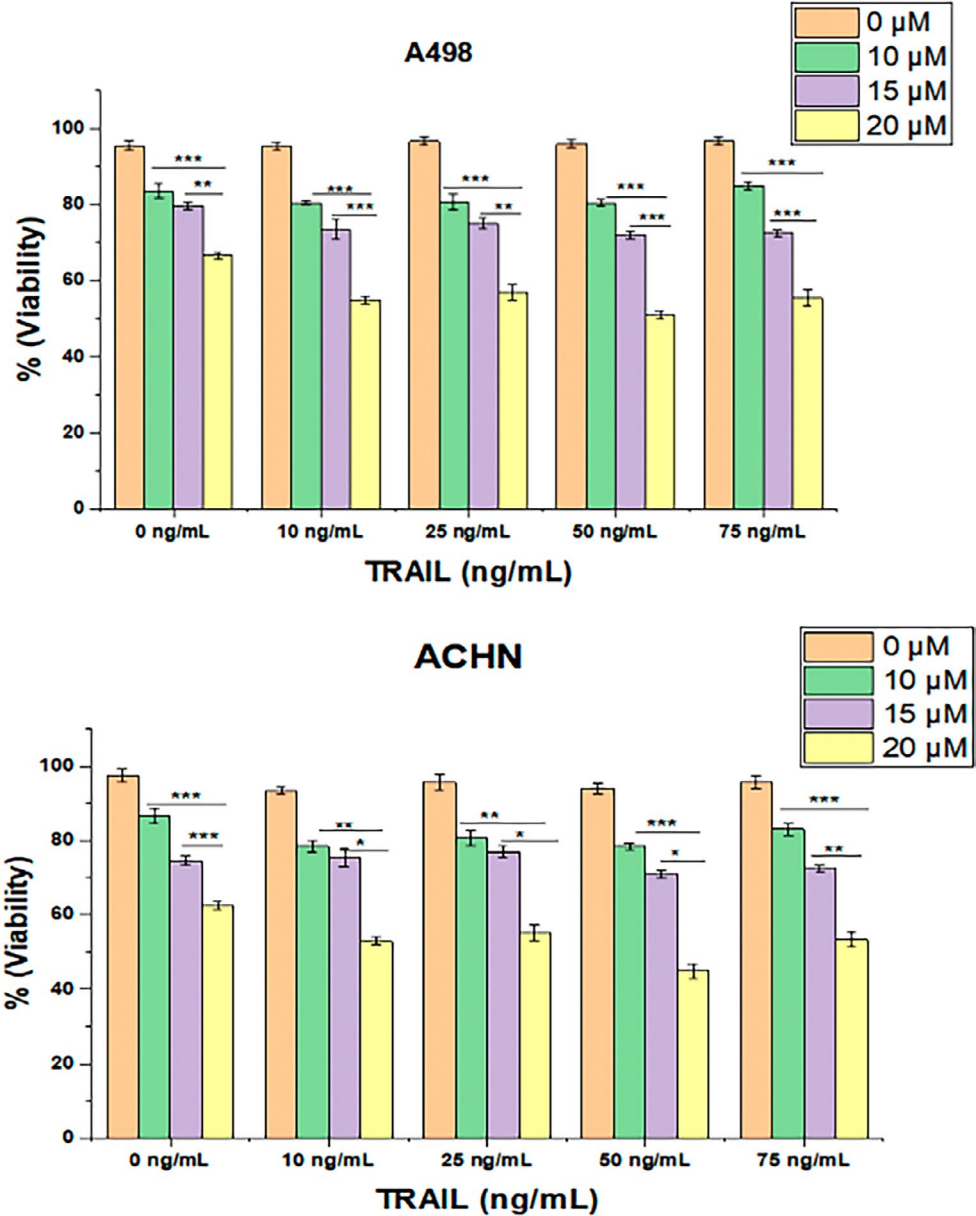
Cell viability assessment of A498 and ACHN after treatment with different concentrations of chrysoeriol and TRAIL. (A) Cell lines were treated with chrysoeriol (0, 10, 15, 20 μM) 5 h before exposure to TRAIL (0, 10, 25, 50, 75 ng/mL) until 24 h. Viability results are expressed as standard deviation of three independent experiment with *, **, and *** indicates significant differences at *p* > 0.05, 0.01, and 0.001, respectively. (B) Regarding synergistic interaction and synergy degree, less than 0.5 was determined as higher degree of synergy between chrysoeriol and TRAIL with strong interaction at 20 μM of chrysoeriol and 50 ng/mL of TRAIL, hence selected for subsequent experiments. Cell viability assessment of A498 and ACHN after treatment with different concentrations of chrysoeriol and TRAIL. (A) Cell lines were treated with chrysoeriol (0, 10, 15, 20 μM) 5 h before exposure to TRAIL (0, 10, 25, 50, 75 ng/mL) until 24 h. Viability results are expressed as standard deviation of three independent experiment with *, **, and *** indicates significant differences at *p* > 0.05, 0.01, and 0.001, respectively. (B) Regarding synergistic interaction and synergy degree, less than 0.5 was determined as higher degree of synergy between chrysoeriol and TRAIL with strong interaction at 20 μM of chrysoeriol and 50 ng/mL of TRAIL, hence selected for subsequent experiments.

Determining cytotoxicity is crucial for selecting an optimal dose for inducing apoptosis in cancer cell lines. To establish a reliable dose for subsequent experiments, combination index (CI) methodology was employed to evaluate interactions between various concentrations of chrysoeriol and TRAIL, specifically at the medium or 50% effect level. This approach allowed for quantitative assessment of drug interactions, where a CI value of less than 0.5 indicated strong synergistic effects, while values greater than 0.5 suggested weaker synergistic interactions (Figure 2).

Mechanistically, observed synergy between chrysoeriol and TRAIL can be attributed to a multifaceted enhancement of both extrinsic and intrinsic apoptotic pathways. Chrysoeriol might upregulate death receptors (DR4) on the cell surface, increasing TRAIL binding and activating the extrinsic apoptotic pathway. This activation promoted the formation of the death‐inducing signaling complex (DISC), leading to caspase‐8 activation. Concurrently, chrysoeriol downregulated anti‐apoptotic proteins such as Bcl‐2, Bcl‐xL, and survivin, disrupting the cellular defense against apoptosis (Obaidi et al. 2020; Chae et al. 2020).

Furthermore, chrysoeriol amplified the intrinsic apoptotic pathway by increasing the expression of the pro‐apoptotic protein (Bax), leading to mitochondrial outer membrane permeabilization (MOMP). This resulted in cytochrome C release, which interacted with apoptotic protease activating factor‐1 (Apaf‐1) to form the apoptosome, activating caspase‐9. This dual activation of caspase‐8 (extrinsic) and caspase‐9 (intrinsic) created a positive feedback loop, enhancing the overall apoptotic response and enhancing the synergy (Obaidi et al. 2020; Micucci et al. 2015; Lemke et al. 2014).

In addition, chrysoeriol might suppress NF‐κB signaling, which is known to promote cell survival and inhibit apoptosis in cancer. By blocking NF‐κB nuclear translocation, chrysoeriol reduced the expression of survival‐promoting genes, sensitizing the cells to TRAIL‐induced apoptosis. The compound also increased ROS generation, causing oxidative stress, further promoting mitochondrial dysfunction and reinforcing apoptotic signaling (Chae et al. 2020; Jeon 2017).

Through careful evaluation of different dose combinations, the combination of 20 μM chrysoeriol and 50 ng/mL TRAIL demonstrated a strong synergistic interaction, consistently yielding CI values below 0.5 in both A498 and ACHN cell lines (Figure 2). Further analysis of cell viability at these concentrations confirmed that the 20 μM chrysoeriol/50 ng/mL TRAIL combination exhibited significantly (*p* < 0.001) lower cytotoxicity while maintaining satisfactory cellular viability. Meanwhile, our results were in accordance with the previously published study (Obaidi et al. 2020), where authors used curcumin and TRAIL synergy on ACHN cell viability. Hence, this combination was, therefore, selected for all subsequent experiments due to its optimal balance of strong apoptotic effect and minimal toxicity.

### 3.2 Morphological Changes in Cell Lines After Treatment

**FIGURE 3:**
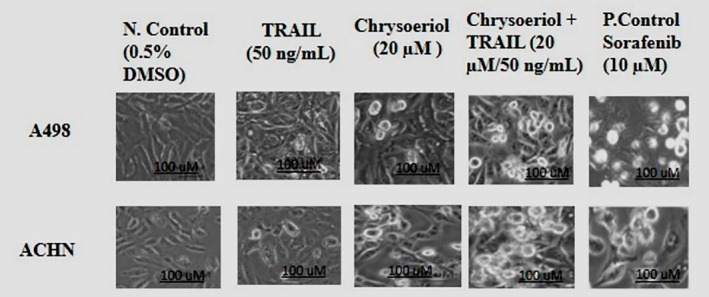
Morphological changes in cell lines after treatment with chrysoeriol and TRAIL. Cells were clearly distorted by synergistic treatment of 20 μM chrysoeriol/50 ng/mL TRAIL. However, the response of A498 cells toward synergistic treatment seemed more evident than ACHN cells due to their higher resistance toward TRAIL, but still the synergistic treatment has made significant distortions and apoptosis in ACHN cells, defining the efficiency of synergistic treatment for apoptosis of both cell lines. Morphological changes in cell lines after treatment with chrysoeriol and TRAIL. Cells were clearly distorted by synergistic treatment of 20 μM chrysoeriol/50 ng/mL TRAIL. However, the response of A498 cells toward synergistic treatment seemed more evident than ACHN cells due to their higher resistance toward TRAIL, but still the synergistic treatment has made significant distortions and apoptosis in ACHN cells, defining the efficiency of synergistic treatment for apoptosis of both cell lines.

Morphology of A498 and ACHN cell lines used in this research was studied by fluorescence microscope after cells treatment with 0.05% DMSO in culture medium as a negative control, 20 μM chrysoeriol, 50 ng/mL TRAIL, and 20 μM chrysoeriol/50 ng/mL TRAIL along with sorafenib as a positive control to confirm positive effects of these treatments on cells apoptosis. Results indicated that treatment solely with TRAIL did not provide significant morphological changes in the cells; however, its combination with chrysoeriol has remarkably increased TRAIL sensitivity for cell lines apoptosis. Approximately 85% apoptosis was observed in 20 μM chrysoeriol/50 ng/mL TRAIL combined dose treatment than solely chrysoeriol and TRAIL treatment (Figure 3).

Moreover, the response of A498 cells towards chrysoetiol/TRAIL treatment was relatively better than that of ACHN cell lines, even with the positive control. ACHN cells are previously reported as highly resistant towards TRAIL treatment (Nalli et al. 2018); however, the TRAIL/chrysoetiol combination has remarkably increased ACHN sensitivity towards treatment. Moreover, the deformation of cells and their bright and elongated shape from the corners indicated cell death due to apoptosis. Their conformational deformation was also observed as a dose‐dependent effect (Figure 3). In addition, untreated cells appeared normal, with a polygonal structural formation and no damage from the corners. However, apoptosis is featured by cell shrinkage, chromatin condensation, and changes in the shape of cells, which is shown to be linked with DNA cleavage (Liang et al. 2020). Hence, our study has clearly indicated that only cells treated with chrysoetiol/TRAIL were damaged properly by the action of this treatment and evidenced the effect of these therapeutic treatments on the apoptosis of renal cancer cells.

Our results aligned with previously published studies on curcumin/TRAIL (Obaidi et al. 2020), Volasertib/TRAIL (Jeon 2017), and withanolide E/TRAIL (Henrich et al. 2015), which reported morphological changes in RCC cell lines. However, these studies were limited to a single cell line, ACHN, which is known for its strong resistance to TRAIL, thus restricting the scope of their findings. Additionally, the concentrations used in these studies were relatively high, raising concerns about cytotoxicity and resulting in significant cellular damage.

In contrast, our study simultaneously evaluated two TRAIL‐resistant RCC cell lines, providing more comprehensive and reliable insights into morphological changes. Notably, we achieved these results using significantly lower treatment concentrations, minimizing cytotoxic effects.

### 3.3 Induction of Cellular Apoptosis in Cell Lines

**FIGURE 4:**
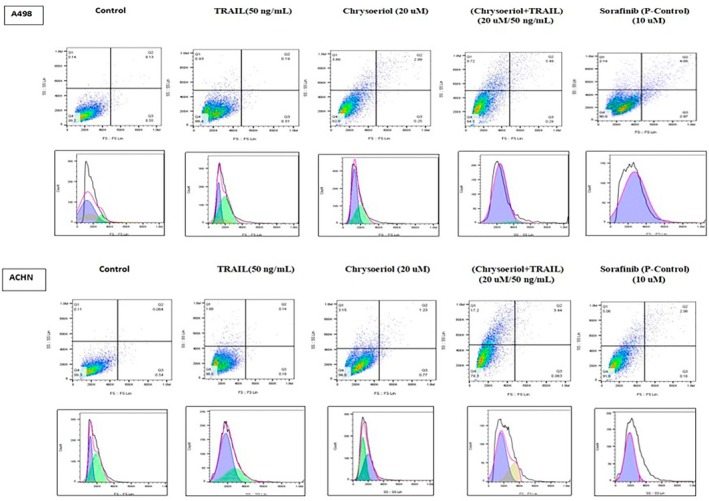
Flow cytometry and mitosis results after treatment. (A) Flow cytometry and cellular phases of mitosis in A498 cell lines after treatments. A498 cells treated with a synergistic combination (20 μM chrysoeriol/50 ng/mL TRAIL) were apoptosed more than with solely TRAIL treatment which indicated that chrysoeriol sensitized TRAIL for the apoptosis of both A498 cells. Treatment with 20 μM chrysoeriol/50 ng/mL TRAIL remarkably decreased cell proliferation by arresting the G1 and S phases; hence, decreased live cell counts indicated cellular apoptosis. (B) Flow cytometry and cellular phases of mitosis in ACHN cell lines after treatments. ACHN cells treated with a synergistic combination (20 μM chrysoeriol/50 ng/mL TRAIL) were apoptosed more than solely treatment with TRAIL, which indicated that chrysoeriol sensitized TRAIL for apoptosis of ACHN cells. Treatment with 20 μM chrysoeriol/50 ng/mL TRAIL remarkably decreased cell proliferation by arresting G1 and S phases; hence, decreased live cell counts indicated cellular apoptosis. Flow cytometry and mitosis results after treatment. (A) Flow cytometry and cellular phases of mitosis in A498 cell lines after treatments. A498 cells treated with a synergistic combination (20 μM chrysoeriol/50 ng/mL TRAIL) were apoptosed more than with solely TRAIL treatment which indicated that chrysoeriol sensitized TRAIL for the apoptosis of both A498 cells. Treatment with 20 μM chrysoeriol/50 ng/mL TRAIL remarkably decreased cell proliferation by arresting the G1 and S phases; hence, decreased live cell counts indicated cellular apoptosis. (B) Flow cytometry and cellular phases of mitosis in ACHN cell lines after treatments. ACHN cells treated with a synergistic combination (20 μM chrysoeriol/50 ng/mL TRAIL) were apoptosed more than solely treatment with TRAIL, which indicated that chrysoeriol sensitized TRAIL for apoptosis of ACHN cells. Treatment with 20 μM chrysoeriol/50 ng/mL TRAIL remarkably decreased cell proliferation by arresting G1 and S phases; hence, decreased live cell counts indicated cellular apoptosis.

Cell lines treated with 0.05% DMSO in culture medium as a negative control, 20 μM chrysoeriol, 50 ng/mL TRAIL, and 20 μM chrysoeriol/50 ng/mL TRAIL along with sorafenib as a positive control were deeply investigated for cellular apoptosis. Annexin V used for cell staining could selectively bind with phosphatidylserine, which usually redistributed outside the cell membrane in early stages of apoptosis (Jeon 2017). After careful consideration of results obtained from cell sorting, dose‐dependent apoptotic events were seen in four gated graphs indicating the presence of viable cells in the lower left corner (Figure 4A,B). Meanwhile, events of early and late apoptosis were characterized by DNA fragmentation after caspase pathway activation, shown in the lower right and upper gate corners, respectively. Moreover, very clear cell sorting has shown that cells treated with a combined synergistic dose of 20 μM chrysoeriol/50 ng/mL TRAIL have shown a higher rate of apoptosis than controls in both cell lines (A498 and ACHN) (Figure 4A,B).

Moreover, apoptosis of cells treated with solely 20 μM chrysoeriol and 50 ng/mL TRAIL has also shown significant differences in results between treatments applied on both cell lines, which was totally matched with results of the cellular morphology section. Meanwhile, both cell lines responded differently towards applied treatments, denoting differences in their sensitivity towards different doses. These results are consistent with previous studies (Min et al. 2020; Wei et al. 2019; Nalli et al. 2018), where different cell lines also responded differently towards different applied treatments for apoptosis of cancer cells. Moreover, ACHN cell lines were found more resistant towards all applied treatments with late apoptosis rates of 1.23 for chrysoeriol and 0.14 for TRAIL; however, still, the synergistic combination of 20 μM chrysoeriol/50 ng/mL TRAIL has increased its sensitivity towards treatments, and programmed cell death in late apoptosis approached a rate of 3.44 in Q2. A498 cell lines responded well towards all treatments, and significant apoptosis of cell lines was examined in the late apoptosis with a rate of 5.49 in Q2 by applying a combined treatment of 20 μM chrysoeriol/50 ng/mL TRAIL. Hence, our study has shown very comprehensive and detailed apoptosis results of all treatments by getting different rates of apoptosis, which is consistent with previously published articles (Obaidi et al. 2020; Jeon 2017; Hassanzadeh et al. 2018; Dimberg et al. 2013) that only elaborated on general apoptotic events.

Afterwards, cell lines were also screened for evaluating the effect of treatments on mitotic phases including G1 (growth phase) indicated by purple color, G2 (cell replenishing phase for synthesizing protein for chromatin manipulation phase) indicated by green color, and S (DNA synthesis phase) indicated by skin color in the pictorial representation (Figure 4A,B).

Cells counts involving G1, G2, and S phases were found to be gradually lowering down with different treatments as compared with control cell lines. It indicated that the applied treatments remarkably affected cell proliferation and survival. However, these results were not shown in previous studies (Obaidi et al. 2020; Chae et al. 2020; Jeon 2017; Henrich et al. 2015) because these authors did not investigate apoptosis at the level of cellular mitosis, hence indicating that this study is a comprehensive study on cellular apoptosis. Moreover, in the control cell lines, all cellular phases could be seen very clearly including G1, G2, and S phases, indicating alive cells in the culture medium full of energy and life. However, treatment with 20 μM chrysoeriol and 50 ng/mL TRAIL affected cell mitosis, with only a few cells remaining in sub G2 and S phase in A498 and ACHN. However, the effect of the combined treatment of 20 μM chrysoeriol/50 ng/mL TRAIL was even more adverse, and only cells were found in sub G1 phase, indicating the apoptosis events have totally deformed cells to further multiply.

### 3.4 Effect of Post Combination Treatment on Activation of Caspases

**FIGURE 5:**
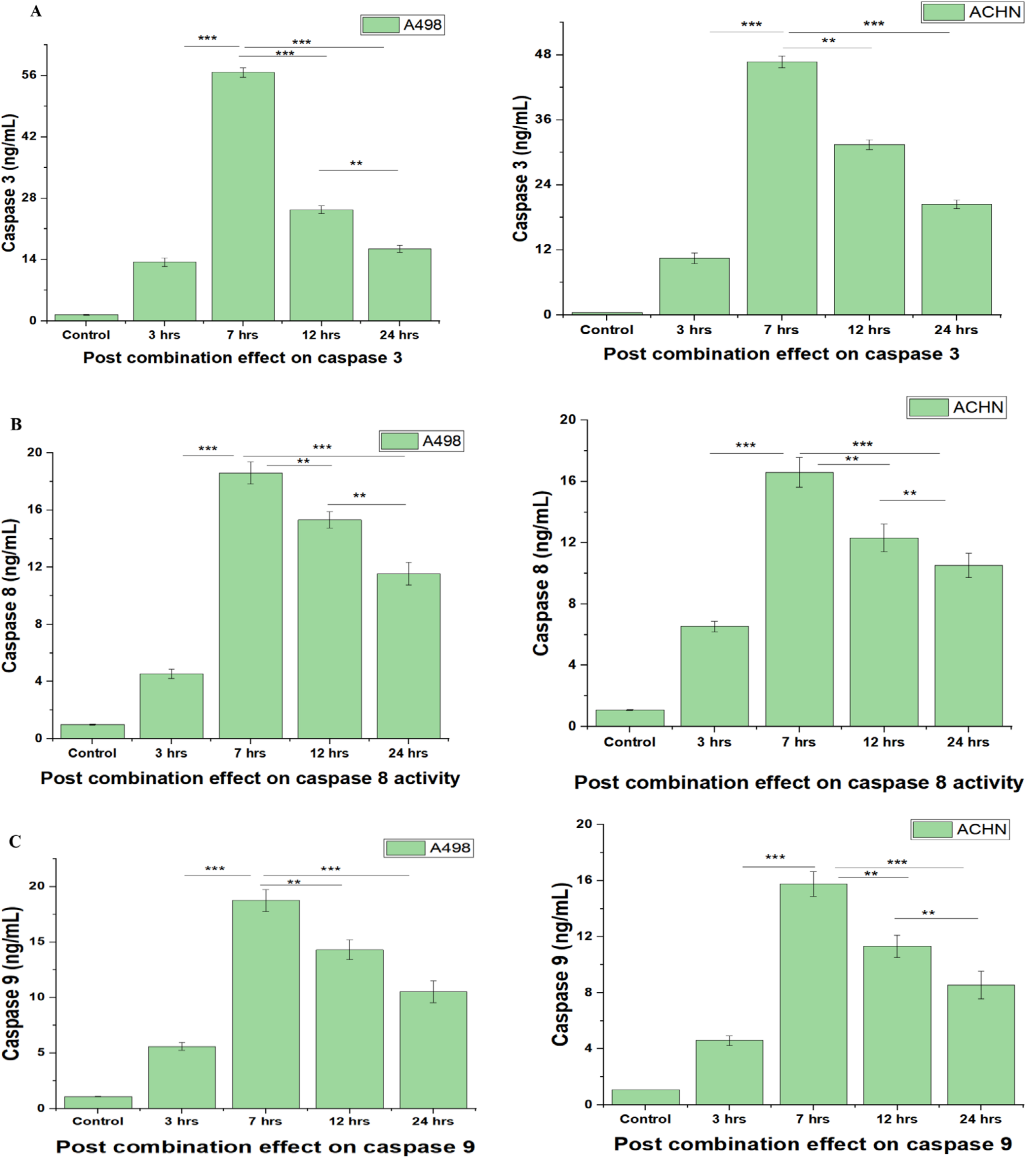
Post combination effect on caspases levels. (A) Post combination effect on caspase‐3 levels. Caspase‐3 levels in both cell lines were increased up to maximum post 7 h treatment with 20 μM chrysoeriol and 50 ng/mL TRAIL; however, a gradual decline in caspase‐3 level was observed after 7 h till 24 h in both cell lines, however with different measurement values. Data represents significant differences at *p* < 0.01 indicated with ** and *p* < 0.001 indicated with ***. (B) Post combination effect on caspase‐8 levels. Caspase‐8 levels in both cell lines were increased up to maximum post 7 h treatment with 20 μM chrysoeriol and 50 ng/mL TRAIL, however, gradual decline in caspase‐8 level was observed after 7 h till 24 h in both cells with different measurement values. Data represents significant differences at *p* < 0.01 indicated with ** and *p* < 0.001 indicated with ***. (C) Post combination effect on caspase‐9 levels. Caspase‐9 levels in both cell lines were increased up to maximum post 7 h treatment with 20 μM chrysoeriol and 50 ng/mL TRAIL, however, gradual decline in caspase‐9 level was observed after 7 h till 24 h in both cell lines, however with different measurement values. Data represents significant differences at *p* < 0.01 indicated with ** and *p* < 0.001 indicated with ***.

Caspases are basically termed as cysteine protease family as well as activated cysteine aspartic proteases, cutting the substrate on specific aspartic acid residues for triggering apoptosis in cells (Kim et al. 2019). Caspases usually act as initiators (caspase‐3) and executioners (caspase‐8, caspase‐9) of apoptosis, evidenced in previous studies (Kim et al. 2019). Meanwhile, different signaling pathways, that is, activators of transcription (JAK/STAT), Bcl‐2/Bax, and FOXO1 could involve caspases as their integral proteases to trigger cellular apoptosis (Kim et al. 2019; Baek et al. 2016). Therefore, caspase‐3 (Figure 5A), caspase‐8 (Figure 5B) and caspase‐9 (Figure 5C) assays were performed to deeply investigate the effect of combined treatments (20 μM chrysoeriol and 50 ng/mL TRAIL) on caspase expression for induction of apoptosis. All caspases were determined to be highly expressed post 7 h of treatment in both cell lines (A498 and ACHN).

However, caspase expression was found significantly (0.01 and 0.001) decreased after 7 h till 24 h in both cell lines, denoting that caspase‐3, caspase‐8, and caspase‐9 were expressed up to maximum limit for cellular apoptosis post 7 h combination treatment. Our results were consistent with a previously published study (Obaidi et al. 2020), which also demonstrated similar findings for caspase‐3, caspase‐8, and caspase‐9 activation across different time intervals within a 24‐h period. However, other studies (Dimberg et al. 2013; Henrich et al. 2015) primarily assessed caspase expression at a single time point using one treatment, focusing solely on obtaining upregulated caspase results. This limited approach not only restricted the understanding of caspase dynamics but also reduced the generalizability of the findings, especially as these studies used only one cell line (Obaidi et al. 2020; Dimberg et al. 2013; Henrich et al. 2015).

In contrast, our study provided a more comprehensive analysis by evaluating caspase activation at multiple time points and using different treatments, offering a broader understanding of apoptotic mechanisms. Notably, we observed that caspase levels were generally lower in ACHN cells than in A498 cells, likely due to ACHN's known resistance to TRAIL. However, the combination of TRAIL with chrysoeriol significantly enhanced caspase activity in ACHN cells, suggesting that chrysoeriol increased TRAIL sensitivity, effectively triggering apoptosis (Chae et al. 2020; Jeon 2017; Hassanzadeh et al. 2018).

### 3.5 Effect of Post Combination Treatment on Regulation of Interleukins

Interleukin's (ILs) are tumor‐promoting cytokines, found in abnormally higher concentrations (particularly IL‐6) in cancer cells, such as renal carcinoma cells (Kamińska et al. 2015). Therefore, their role in the proliferation of cancer cells is evident by their invasion, migration, and malignant behaviors by different mechanisms in cancer pathology (Bharti et al. 2016). Meanwhile, IL‐6 regulation is more highlighted in cancer pathology due to its increased signal activation and great contribution to micro‐environment formation, hence triggering the initiation and progression of tumors (Kamińska et al. 2015). Meanwhile, IL‐10 is an antagonist to IL‐6 and is termed an immunosuppressive cytokine, inducing anti‐cancer effects while its activation promotes tumor cell apoptosis (Qiao et al. 2019). A relatively lower expression of IL‐10 can cause inflammation as well as cancer proliferation (Wang et al. 2016). Therefore, it is important to deeply investigate the regulation of both IL‐6 and IL‐10 during the treatment of cancer cells. In this study, post‐combination treatment effects on both IL‐6 and IL‐10 are investigated after different hours of treatment.

**FIGURE 6:**
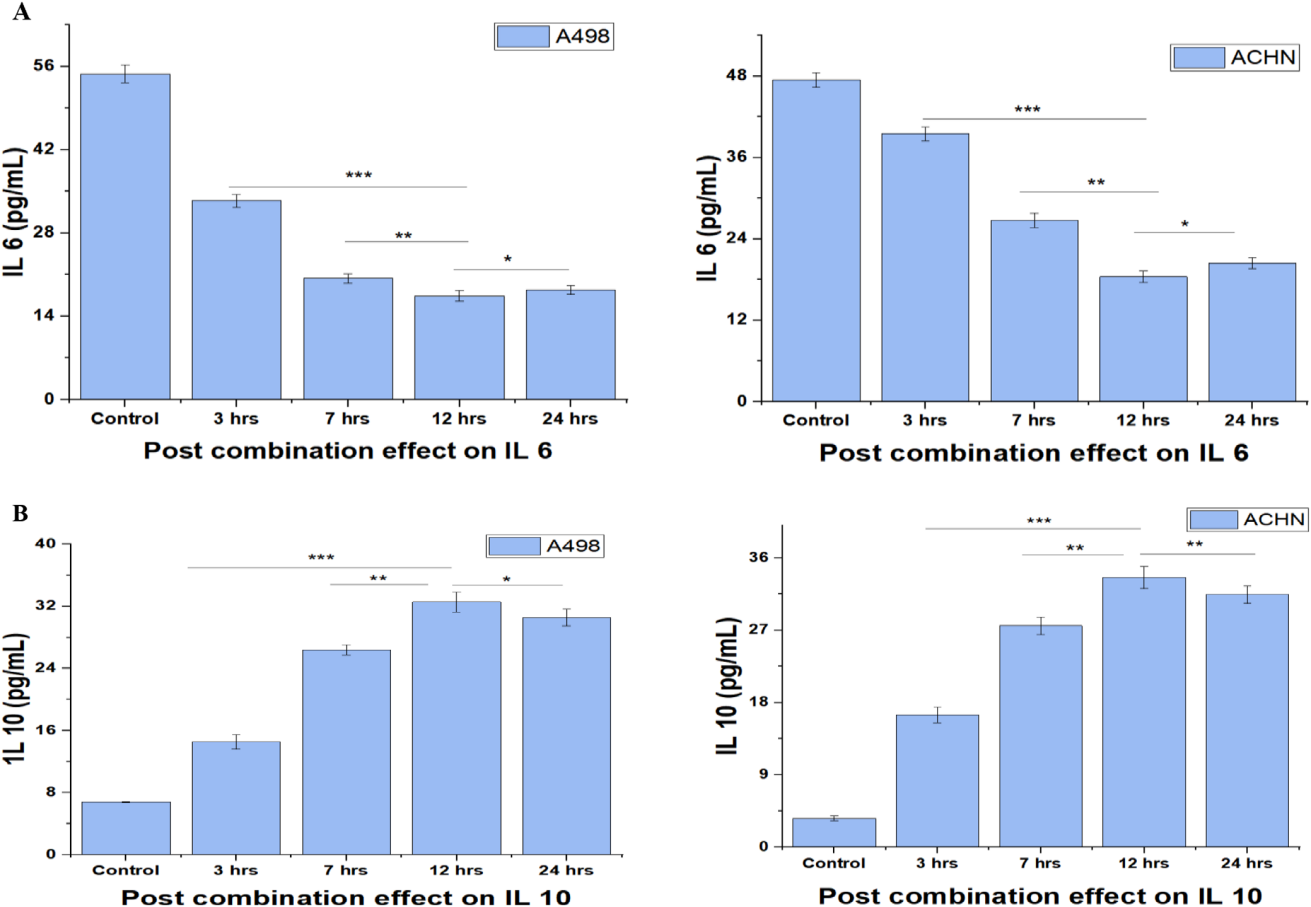
Post combination effect on ILs levels. (A) Post combination effect on IL‐6 levels. IL‐6 levels in both cell lines were significantly (*p* < 0.01, *p* < 0.001, *p* < 0.05) decreased post 7 to 12 h treatment with 20 M chrysoeriol and 50 ng/mL TRAIL; however, a gradual rise (*p* < 0.05) in IL‐6 level was observed after 12 h till 24 h in both cell lines, albeit with different measurement values. Data represent significant differences at *p* < 0.05 indicated with *, *p* < 0.01 indicated with **, and *p* < 0.001 indicated with ***. (B) Post combination effect on IL‐10 levels. IL‐6 levels in both cell lines were significantly (*p* < 0.01, *p* < 0.001, *p* < 0.05) increased post 7 to 12 h treatment with 20 M chrysoeriol and 50 ng/mL TRAIL; however, a gradual decline (*p* < 0.05, *p* < 0.01) in IL‐10 level was observed after 12 h till 24 h in both cell lines, albeit with different measurement values. Data represent significant differences at *p* < 0.05 indicated with *, *p* < 0.01 indicated with ** and *p* < 0.001 indicated with ***.

However, an interesting piece of information was obtained by carefully examining that significant lowering of IL‐6 expression was observed during 7 to 12 h post treatment (Figure 6A). After 12 h, IL‐6 expression was observed to be increased again till 24 h. It indicated that the best effect of post combination treatment was obtained between 7 to 12 h for IL‐6. Linking IL‐6 down‐regulation with up‐regulation of IL‐10, the same trend seemed reversed by up‐regulation of IL‐10 (Figure 6B) during 7 to 12 h post combination treatment. This effect indicated that apoptosis of renal cell lines (A498 and ACHN) occurred during 7 to 12 h of treatment (IL‐6 down‐regulated and IL‐10 up‐regulated).

Moreover, our findings aligned with a previously published study (Batool et al. 2021) that reported a significant reduction in IL‐6 expression and an upregulation of IL‐10 following treatment with a natural medicine. However, we only assessed IL‐6 and IL‐10 expression at a single time point (24 h); in contrast, our study systematically monitored their expression at 3, 7, 12, and 24 h, providing a detailed temporal profile of their modulation and an understanding of their behavior under treatment.

### 3.6 Effect of Treatment on Regulation of Proteosome Activity

**FIGURE 7:**
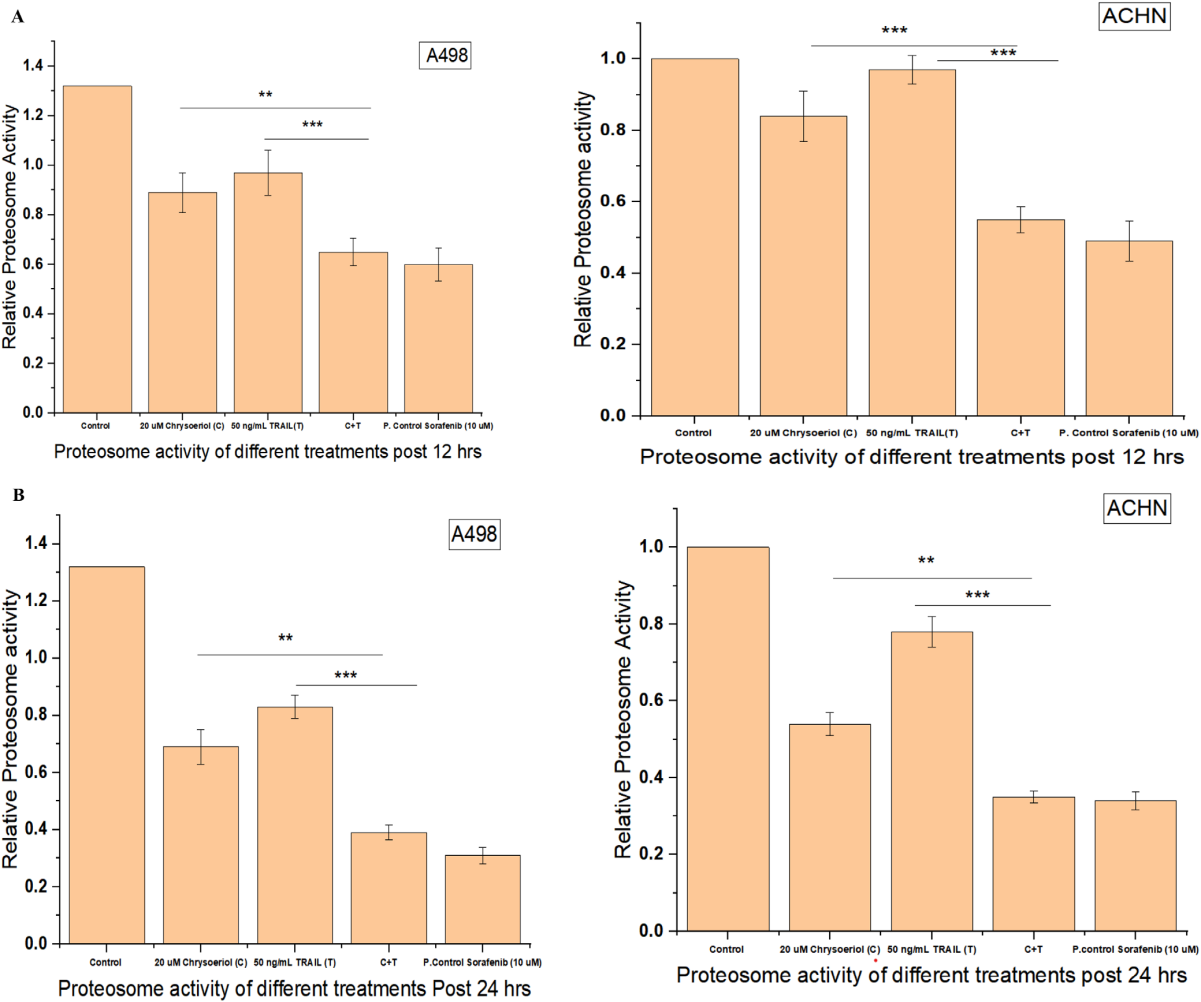
Proteasome activity. 7A Inhibition of proteasome activity by applying different treatments in A498 and ACHN post 12 h. Proteasome activity was significantly (*p* < 0.01, *p* < 0.001) decreased by the combined synergistic treatment of 20 μM chrysoeriol and 50 ng/mL TRAIL compared to TRAIL alone, indicating sensitization of TRAIL with chrysoeriol post 12 h. Data represent significant differences at *p* < 0.01 indicated with ** and *p* < 0.001 indicated with ***. (B) Inhibition of proteasome activity by applying different treatments in A498 and ACHN post 24 h. Proteasome activity was significantly (*p* < 0.01, *p* < 0.001) decreased by the combined synergistic treatment of 20 μM chrysoeriol and 50 ng/mL TRAIL compared to TRAIL alone, indicating the sensitization of TRAIL with chrysoeriol post 24 h. This indicated that increased treatment has a positive effect on the decrement of proteasome activity. Data represent significant differences at *p* < 0.01 indicated with ** and *p* < 0.001 indicated with ***.

Chymotrypsin‐like proteasome activity is an important activity to be considered for evaluating protein degradation, as well as highly attractive for anti‐cancer drug therapy due to the strong role of proteasomes in carcinogenesis (Voutsadakis 2017). Targeting proteasomes inhibited the proliferation of cancer cells by the induction of apoptosis evidenced in vitro and in vivo (Banerjee et al. 2018). Therefore, this study also focused on evaluating proteasome activity deeply by treating both cell lines separately with 20 μM chrysoeriol, 50 ng/mL TRAIL, and their synergistic combination (20 μM chrysoeriol and 50 ng/mL TRAIL) and investigated their effects after 12 (Figure 7A) and 24 (Figure 7B) hours post treatment. Maximum inhibition of proteasome activity was obtained post 24 h treatment with the synergistic combination of 20 μM chrysoeriol and 50 ng/mL TRAIL in A498 and ACHN cells. However, proteasome activity was inhibited more in A498 than in ACHN cells, indicating the relative resistance of ACHN towards this treatment. However, still, ACHN cells have given a good response towards synergistic treatment by significantly decreasing proteasome activity post 24 h of treatment (Figure 7B). Meanwhile, treatment solely with TRAIL failed to inhibit the chymotryptic 20S subunit of the proteasome. However, treatment solely with chrysoeriol inhibited proteasome activity, indicating the response of cell lines towards chrysoeriol treatment.

Meanwhile, our findings were aligned with previous studies demonstrating the inhibitory effects of the natural compound curcumin (a flavonoid) on the chymotryptic activity of proteasomes in cancer cells (Obaidi et al. 2020; Banerjee et al. 2018; Khan et al. 2018). Study (Obaidi et al. 2020) received very consistent results with our study by achieving inhibition of proteasome activity post 24 h treatment with curcumin on ACHN cells. Furthermore, study (Banerjee et al. 2018) reported that curcumin impaired 26S proteasome activity by directly inhibiting dual‐specificity tyrosine‐regulated kinase 2, while study (Khan et al. 2018) showed curcumin‐mediated degradation of S‐phase kinase protein 2, triggering cytotoxicity in both human papillomavirus‐positive and negative squamous carcinoma cells.

### 3.7 Effect of Synergistic Treatments on Mitochondrial Membrane Potential (MMP) and Cytochrome C Release

**FIGURE 8:**
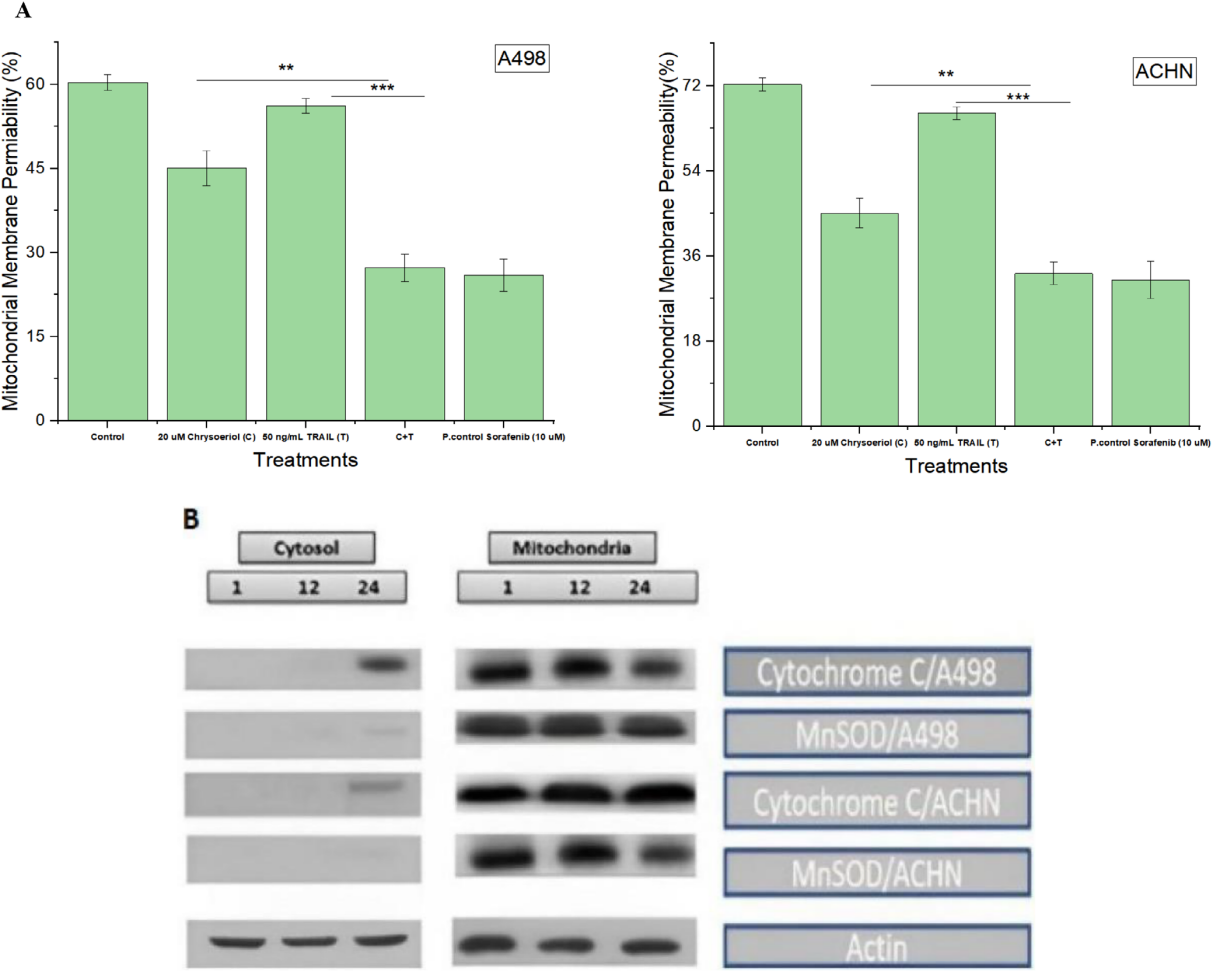
Mitochondrial membrane potential and cytochrome C release. (A) Loss of mitochondrial membrane potential after synergistic treatment on A498 and ACHN. Synergistic treatment with 20 μM chrysoeriol and 50 ng/mL TRAIL caused a remarkable loss in MMP in both cell lines, indicating the triggering of apoptosis. Data represent significant differences at *p* < 0.01 indicated with ** and *p* < 0.001 indicated with ***. (B) Cytochrome C release from mitochondria in the cytoplasm after the treatment of cell lines with the subsequent synergistic treatments. Western blot results indicated treatment effects after 1, 12, and 24 h time periods in the cytosol and mitochondria of both cell lines. However, cytochrome C release was more prominent in the A498 cell line than in ACHN cells at 24 h.

Mitochondrial membrane potential (MMP) is a key factor for inducing apoptosis in cancer cells due to its direct relation with oxidative stress. Loss or gain of MMP is directly linked with apoptosis or proliferation of cancer cells, respectively. Therefore, this study explored the effects of 20 μM chrysoeriol, 50 ng/mL TRAIL, and their synergistic effect on loss of MMP in A498 and ACHN cells by using rhodamine 123 fluorescent dye. We have observed that MMP loss was linked to synergistic treatment‐induced apoptosis of the cell lines. Synergistic treatment‐treated cell lines have shown reduced MMP. However, this loss was more prominent in the A498 cell line than ACHN cells (Figure 8A).

Our findings aligned with previously reported studies demonstrating the inhibition of MMP by niclosamide in renal carcinoma cells (Zhao et al. 2016) and curcumin in human squamous carcinoma cells within 24 h of treatment (Khan et al. 2018). However, these studies primarily focused on single treatments without exploring potential synergistic effects of combined therapies. In contrast, our study uniquely investigated the combined effects of chrysoeriol and TRAIL on MMP inhibition, providing a more comprehensive understanding of their synergistic potential. This approach not only enhanced the inhibitory effects on MMP but also offered deeper insights into their combined therapeutic potential, surpassing the scope of the previous studies.

Moreover, we further investigated mitochondrial cytochrome C release in cell lines treated with a synergistic combination of chrysoeriol and TRAIL, a critical indicator of apoptosis or proliferation in renal cancer cells. Our results clearly demonstrated that cytochrome C was released from mitochondria into the cytoplasm following treatment, signifying that cells were undergoing apoptosis. The effects of treatment were assessed at 1 h, 12 h, and 24 h intervals in the cytosol and mitochondria of both cell lines. Notably, after 24 h, a more pronounced cytochrome C release was observed in A498 cells compared to ACHN cells (Figure 8B), indicating a higher susceptibility of A498 cells to apoptosis. This finding highlighted the differential sensitivity of renal cancer cell lines to the synergistic treatment. Our study provided a comprehensive understanding of the synergistic effects of chrysoeriol and TRAIL on MMP disruption and cytochrome C release, which were not explored in previous studies (Obaidi et al. 2020; Banerjee et al. 2018) or limited research was carried out by giving only single treatment effects within 24 h on cytochrome C release (Khan et al. 2018). This added a novel aspect to our research by elucidating the enhanced apoptotic mechanisms triggered by the combined treatment.

### 3.8 Effect of Different Treatments on Death Receptor 4 (DR4) Expression

**FIGURE 9:**
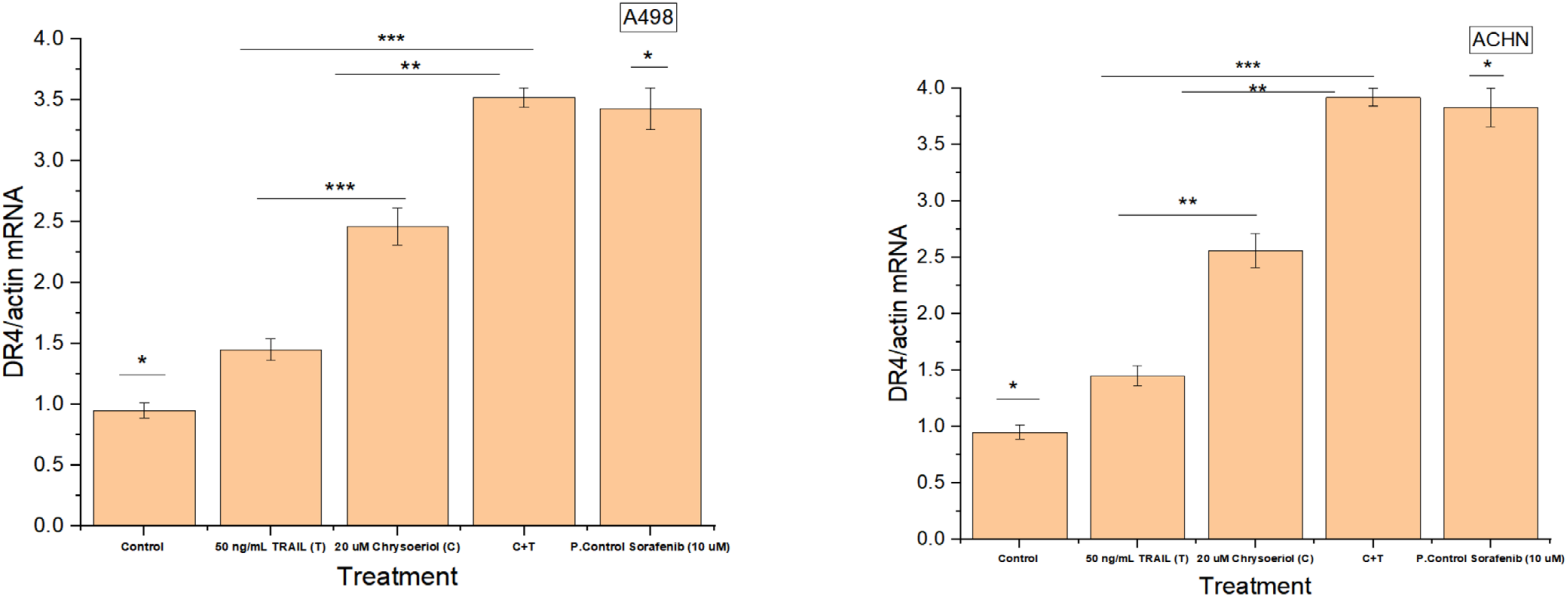
DR4 mRNA expression after treatment with 20 μM chrysoeriol, 50 ng/mL TRAIL, and their synergistic combination (20 μM chrysoeriol and 50 ng/mL TRAIL). Significant (*p* < 0.01, *p* < 0.001) rise in the expression of DR4 was obtained by synergistic combination treatment than treatments solely with TRAIL, indicating sensitization of TRAIL with chrysoeriol for up‐regulating DR4 expression in both cell lines. Data represent significant differences at *p* < 0.01 indicated with ** and *p* < 0.001 indicated with ***.

Death receptor 4 is an important cell surface receptor mediating apoptosis or leading to the secretion of inflammatory cytokines by binding its ligands (Zhang et al. 2021). Our study hypothesized that chrysoeriol could affect DR4 expression of TRAIL for its sensitization to trigger the apoptosis of renal carcinoma cell lines. To explore this, we examined DR4 expression following treatments with 20 μM chrysoeriol, 50 ng/mL TRAIL, and their synergistic combination (20 μM chrysoeriol and 50 ng/mL TRAIL). Our results demonstrated a significant (*p* < 0.01, *p* < 0.001) increase in DR4 expression with synergistic treatment compared to TRAIL alone (Figure 9). This aligned with findings from a previous study (Obaidi et al. 2020), where DR4 expression was also enhanced using a curcumin and TRAIL combination. However, our study differed in several key aspects. First, while the previous study used curcumin, we employed chrysoeriol at optimized concentrations. Second, their investigation was limited to ACHN cells, whereas we assessed DR4 expression in two renal cancer cell lines (A498 and ACHN), expanding the scope of our findings. Notably, our study demonstrated a two‐fold higher DR4 expression than the previous study, indicating that our optimized synergistic treatment was more effective in enhancing DR4 expression to promote apoptosis. This highlighted the superior efficacy of chrysoeriol and TRAIL synergy in modulating apoptosis through DR4 activation. Meanwhile, treatment with TRAIL provided relatively lower expression of DR4 than treatment with chrysoeriol, hence indicating that chrysoeriol sensitized TRAIL for expressing DR4 in synergistic treatment, which ultimately led to the apoptosis of cell lines by an enhanced DR4 expression.

### 3.9 Synergistic Treatment Induced the Down‐Regulation and Up‐Regulation of Certain Genes

AKT1 and FOXO1 exhibited opposing roles in cell cycle regulation. FOXO1 regulated a key cell cycle protein, the cyclin‐dependent kinase inhibitor (CKI/p27^kip1) (Dijkers 2000), and mediates apoptosis through factors such as Bim, ligands, and Fas (Tang 2002). Notably, FOXO1 is often downregulated in various cancers, contributing to uncontrolled cell proliferation. Conversely, AKT1 overexpression inhibited FOXO1 activity, particularly suppressing apoptosis in cancer cells, including renal cancer (Altomare et al. 2002). To promote apoptosis in renal cancer cells, reducing AKT1 expression while enhancing FOXO1 expression is critical. Hence, this study specifically examined the effects of AKT1 downregulation and FOXO1 upregulation on inducing apoptosis in A498 and ACHN cells using 20 μM chrysoeriol, 50 ng/mL TRAIL, and their combined synergistic treatment.

**FIGURE 10:**
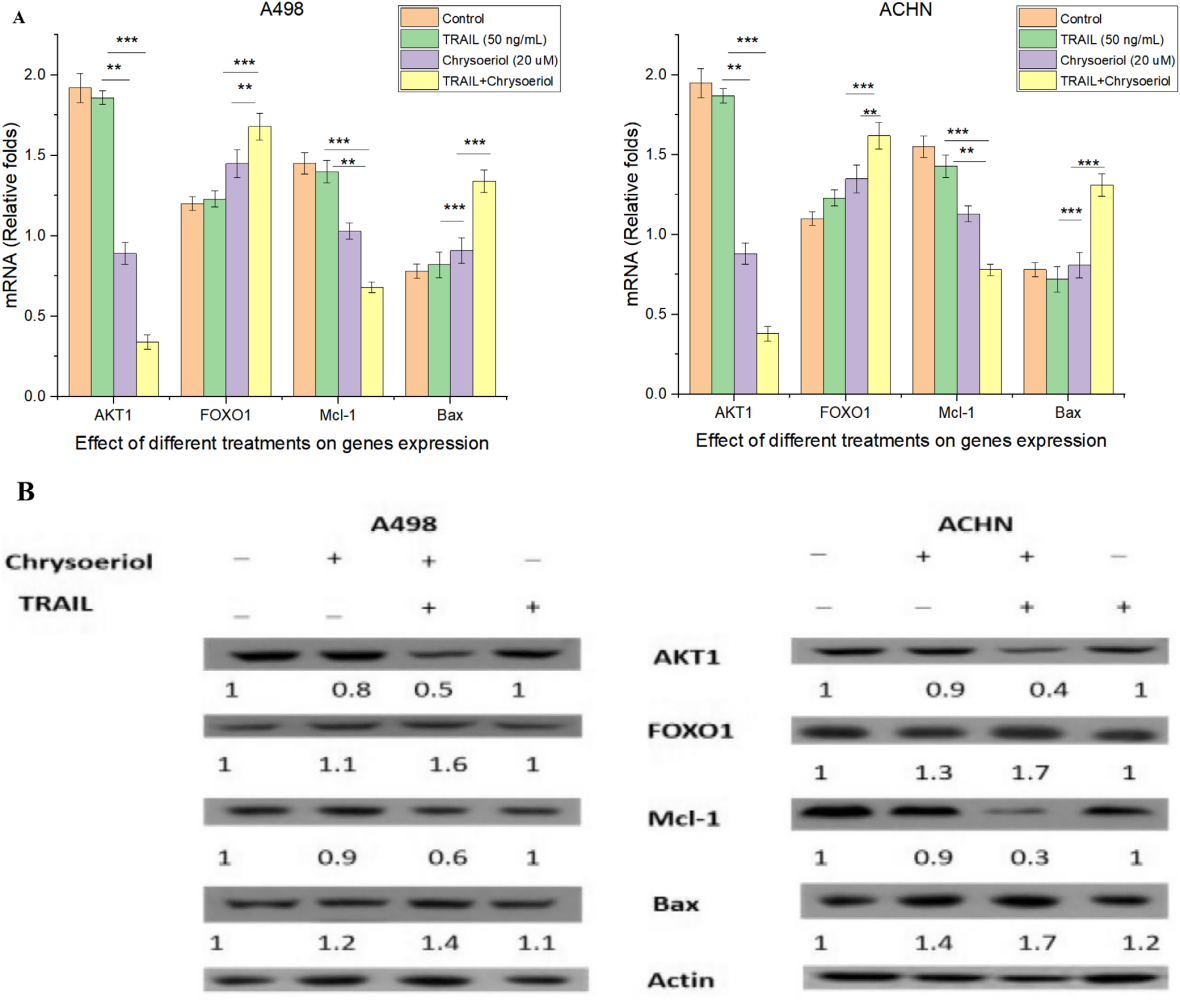
mRNA expression and western blot. (A) Synergistic treatments (20 μM chrysoeriol and 50 ng/mL TRAIL) effect on mRNA expression of anti‐apoptotic and pro‐apoptotic genes in A498 and ACHN cells. Data represents significant differences at *p* < 0.01 indicated with ** and *p* < 0.001 indicated with ***. (B) Western Blot. Western Blot results of synergistic treatments (20 μM chrysoeriol and 50 ng/mL TRAIL) effect on protein expression of anti‐apoptotic and pro‐apoptotic genes in A498 and ACHN cells._ indicated control cell lines with no treatment; however, +indicated synergistic treatment of cells.

AKT1 expression was higher in ACHN control cells than in A498 cells, suggesting that upregulated AKT1 might be associated with greater resistance of ACHN cells to various treatments, including TRAIL. Following synergistic treatment, a significant reduction in AKT1 expression was observed in both cell lines (Figure 10A). Protein expression levels in both cell lines were further analyzed using Western blotting (Figure 10B), which clearly demonstrated the differences in protein expression before and after treatments. Our results demonstrating the downregulation of AKT1 expression were aligned with a previous study (Henrich et al. 2015) that employed withanolide E/TRAIL to induce apoptosis in Caki‐1 and ACHN cells. While the previous study focused solely on protein expression, our research provided comprehensive insights by assessing both protein and RNA expression, offering a deeper understanding of the molecular mechanisms underlying AKT1 regulation. Although Caki‐1 and ACHN cells share morphological similarities, A498 presented distinct characteristics, demonstrating that AKT1 downregulation is not limited to a specific cell type but may be a broader phenomenon in renal cancer cells.

Apoptosis in cancer cells can also be regulated by specific genes categorized into two main groups by the Bcl‐2 protein family: anti‐apoptotic and pro‐apoptotic genes. Anti‐apoptotic genes, including Bcl‐XL, Bcl‐2, and Mcl‐1, inhibit apoptosis, promoting cell survival. In contrast, pro‐apoptotic genes, such as Bok, Hrk, Bax, and Bad, promote apoptosis by activating the apoptotic process (Woo et al. 2020). Changes in the expression of pro‐apoptotic genes can directly influence apoptosis. Bax is a key pro‐apoptotic factor that initiates apoptosis, while Mcl‐1, an anti‐apoptotic factor, supports tumor growth. Notably, Mcl‐1 downregulation indicates apoptosis in cancer cells. This study specifically investigates the expression of Mcl‐1 and Bax to assess how programmed cell death occurs through the mitochondrial pathway in renal carcinomas. Following treatment, Mcl‐1 expression was significantly downregulated in both ACHN and A498 cell lines, consistent with previous studies (Jeon 2017; Henrich et al. 2015; Nalli et al. 2018). Study (Henrich et al. 2015) utilized withanolide E/TRAIL to induce apoptosis in Caki‐1 and ACHN cells by downregulating Mcl‐1 expression, while study (Jeon 2017) employed Volasertib/TRAIL to trigger apoptosis specifically in Caki‐1 cells by downregulating Mcl‐1 expression. However, (Nalli et al. 2018) used rocaglates/TRAIL in ACHN cells to trigger apoptosis. Our study, however, demonstrated the downregulation of Mcl‐1 expression in ACHN and A498 cells using a synergistic combination of chrysoeriol and TRAIL for the very first time and obtained much lower expression of Mcl‐1 by getting both RNA and protein expression altogether than obtained by (Jeon 2017; Henrich et al. 2015; Nalli et al. 2018). This distinction not only highlights the broader applicability of our approach across different renal cancer cell lines but also emphasizes the potential of chrysoeriol/TRAIL as an effective therapeutic strategy against renal carcinoma, providing novel insights beyond those reported in previous studies.

In this study, we further explored the upregulation of Bax and FOXO1 expression in both A498 and ACHN cell lines following synergistic treatment with chrysoeriol/TRAIL. Our findings demonstrated that expression levels of Bax and FOXO1 were significantly higher in A498 cells compared to ACHN cells, as evidenced by both RNA and protein expression analyses. This pronounced upregulation in A498 suggested that ACHN cells might exhibit greater resistance to the synergistic effects of chrysoeriol/TRAIL. Our results for Bax upregulation are consistent with previous research (Obaidi et al. 2020), which reported synergistic effects of curcumin/TRAIL in enhancing Bax protein expression in ACHN cells, and study (Henrich et al. 2015), which demonstrated similar upregulation with withanolide E/TRAIL in ACHN and Caki‐1 cells. However, our study provided a more comprehensive analysis by evaluating both RNA and protein expression of Bax in two distinct RCC cell lines (A498 and ACHN), establishing a more robust understanding of the apoptotic mechanisms triggered by chrysoeriol/TRAIL. This dual‐level analysis highlighted broader efficacy of our approach, offering a clearer perspective on the molecular dynamics of apoptosis in RCC cells. Moreover, our study is the first to report the upregulation of FOXO1 as a key mechanism for inducing apoptosis in renal carcinoma cells following synergistic treatment with chrysoeriol/TRAIL. Our results demonstrated a significant increase in FOXO1 expression at both RNA and protein levels, providing strong evidence that FOXO1 upregulation might serve as a critical factor in triggering apoptosis in renal cancer cells. This novel finding not only expanded the understanding of the apoptotic pathways activated by this synergistic treatment but also highlighted FOXO1 as a potential therapeutic target for enhancing cancer cell susceptibility to apoptosis.

## 4 Conclusion

This study demonstrated the potent chemo‐sensitizing effect of chrysoeriol in enhancing the apoptotic response of TRAIL‐resistant renal carcinoma cells, highlighting the critical role of genetic control in improving TRAIL efficacy. The synergistic combination of chrysoeriol and TRAIL significantly enhanced apoptosis through multiple mechanisms, including increased caspase activation, interleukin modulation, cellular apoptosis, proteasome activity, mitochondrial membrane potential disruption, and cytochrome C release. Notably, this combination treatment led to the up‐regulation of death receptor 4 (DR4) and pro‐apoptotic genes, alongside the down‐regulation of anti‐apoptotic genes, underscoring its robust anti‐cancer potential.

These findings have substantial therapeutic implications, suggesting that targeting specific genetic pathways can improve treatment outcomes in TRAIL‐resistant renal carcinoma. The study provides a strong foundation for developing personalized treatment strategies, where genetic profiling could identify patients most likely to benefit from chrysoeriol‐TRAIL combination therapy. Moreover, it opens avenues for the creation of targeted therapeutics that enhance apoptosis in resistant cancer cells. Nevertheless, further in vivo studies and well‐designed human clinical trials are essential to validate the safety, efficacy, and clinical applicability of this innovative therapeutic approach.

## Author Contributions

**Zahra Batool:** conceptualization (equal), investigation (equal), methodology (equal), validation (equal), writing – original draft (equal), writing – review and editing (equal). **Guobin Weng:** data curation (equal), formal analysis (equal). **Mohammad Amjad Kamal:** data curation (equal), formal analysis (equal). **Qihang Wu:** supervision (equal). **Bairong Shen:** funding acquisition (equal), project administration (equal), resources (equal), supervision (equal).

## Ethics Statement

The authors have nothing to report.

## Conflicts of Interest

The authors declare no conflicts of interest.

## Data Availability

Data will be available on demand.

## References

[fsn370442-bib-0001] Aboulaghras, S. , N. Sahib , S. Bakrim , et al. 2022. “Health Benefits and Pharmacological Aspects of Chrysoeriol.” Pharmaceuticals 15: 973. 10.3390/ph15080973. 36015121 PMC9415049

[fsn370442-bib-0002] Altomare, D. A. , T. Satoshi , D. Assunta , et al. 2002. “Frequent Activation of AKT2 Kinase in Human Pancreatic Carcinomas.” Journal of Cellular Biochemistry 87, no. 4: 470–476. 10.1002/jcb.10287. 14735903

[fsn370442-bib-0003] Baek, H. J. Y. , M. L. Yong , H. K. Tae , et al. 2016. “Caspase‐3/7‐Mediated Cleavage of β2‐Spectrin Is Required for Acetaminophen‐Induced Liver Damage.” International Journal of Biologicsl Science 12, no. 2: 172–183. 10.7150/ijbs.13420 PMC4737674 26884715

[fsn370442-bib-0004] Banerjee, S. , C. Ji , J. E. Mayfield , et al. 2018. “Ancient Drug Curcumin Impedes 26S Proteasome Activity by Direct Inhibition of Dual‐Specificity Tyrosine‐Regulated Kinase 2.” Proceedings of the National Academy of Sciences of the United States of America 115: 8155–8160. 29987021 10.1073/pnas.1806797115 PMC6094102

[fsn370442-bib-0005] Batool, Z. , G. Hu , X. Huang , et al. 2021. “Dietary Therapeutic Treatment of Renal Carcinoma Cell Lines by Down‐Regulating cFlip, Mcl‐1, Bcl‐XL and STAT3 Gene Expression Under the Influence of Up‐Regulated Bax and Intrinsic Apoptotic Pathway.” Food Bioscience 43: 101319.

[fsn370442-bib-0006] Bharti, R. , G. Dey , and M. Mandal . 2016. “Cancer Development, Chemoresistance, Epithelial to Mesenthymal Transition and Stem Cells: A Snapshot of IL‐6 Mediated Involvement.” Cancer Letters 375: 51–61. 26945971 10.1016/j.canlet.2016.02.048

[fsn370442-bib-0007] Chae, I. G. , N. Y. Song , D. H. Kim , M. Y. Lee , J. M. Park , and K. S. Chun . 2020. “Thymoquinone Induces Apoptosis of Human Renal Carcinoma Caki‐1 Cells by Inhibiting JAK2/STAT3 Through Pro‐Oxidant Effect.” Food and Chemical Toxicology 139: 111253. 10.1016/j.fct.2020.111253. 32165235

[fsn370442-bib-0008] Chou, T. C. , and P. Talalay . 1984. “Quantitative Analysis of Dose‐Effect Relationships: The Combined Effects of Multiple Drugs or Enzyme Inhibitors.” Advances in Enzyme Regulation 22: 27–55. 6382953 10.1016/0065-2571(84)90007-4

[fsn370442-bib-0009] Dijkers, P. F. 2000. “Forkhead Transcription Factor FKHR‐L1 Modulates Cytokine‐Dependent Transcriptional Regulation of p27 KIP1.” Molecular and Cellular Biology 20, no. 24: 9138–9148. 10.1128/mcb.20.24.9138-9148.2000. 11094066 PMC102172

[fsn370442-bib-0010] Dimberg, L. Y. , C. K. Anderson , R. Camidge , K. Behbakht , A. Thorburn , and H. L. Ford . 2013. “On the TRAIL to Successful Cancer Therapy? Predicting and Counteracting Resistance Against TRAIL‐Based Therapeutics.” Oncogene 32: 1341–1350. 22580613 10.1038/onc.2012.164 PMC4502956

[fsn370442-bib-0011] Hassanzadeh, A. , M. Farshdousti Hagh , and M. R. Alivand . 2018. “Down‐Regulation of Intracellular Anti‐Apoptotic Proteins, Particularly c‐FLIP by Therapeutic Agents; the Novel View to Overcome Resistance to TRAIL.” Journal of Cellular Physiology 233: 6470–6485. 29741767 10.1002/jcp.26585

[fsn370442-bib-0012] Henrich, C. J. , A. D. Brooks , K. L. Erickson , et al. 2015. “With Anolide E Sensitizes Renal Carcinoma Cells to TRAIL‐Induced Apoptosis by Increasing cFLIP Degradation.” Cell Death & Disease 6: e1666. 25719250 10.1038/cddis.2015.38 PMC4669816

[fsn370442-bib-0013] Huo, L. , C. W. Li , T. H. Huang , et al. 2014. “Activation of keap1/nrf2 Signaling Pathway by Nuclear Epidermal Growth Factor Receptor in Cancer Cells.” American Journal of Translational Research 6, no. 6: 649–663. http://www.ncbi.nlm.nih.gov/pubmed/25628777. 25628777 PMC4297334

[fsn370442-bib-0014] Jeon, M. Y. 2017. “Volasertib Enhances Sensitivity to TRAIL in Renal Carcinoma Caki Cells Through Downregulation of c‐FLIP Expression.” International Journal of Molecular Sciences 18, no. 12: 2568. 10.3390/ijms18122568. 29186071 PMC5751171

[fsn370442-bib-0015] Kamińska, K. , A. M. Czarnecka , B. Escudier , F. Lian , and C. Szczylik . 2015. “Interleukin‐6 as an Emerging Regulator of Renal Cell Cancer.” Urologic Oncology: Seminars and Original Investigations 33, no. 11: 476–485. 10.1016/j.urolonc.2015.07.010. 26296264

[fsn370442-bib-0016] Khan, A. Q. , K. S. Siveen , K. S. Prabhu , et al. 2018. “Curcumin‐Mediated Degradation of S‐Phase Kinase Protein 2 Induces Cytotoxic Effects in Human Papillomavirus‐Positive and Negative Squamous Carcinoma Cells.” Frontiers in Oncology 8: 399. 30333956 10.3389/fonc.2018.00399 PMC6176276

[fsn370442-bib-0017] Kim, B. I. , J. H. Kim , D. Y. Sim , et al. 2019. “Inhibition of JAK2/STAT3 and Activation of Caspase‐9/3 Are Involved in KYS05090S‐Induced Apoptosis in Ovarian Cancer Cells.” International Journal of Oncology 55, no. 1: 203–210. 10.3892/ijo.2019.4795. 31059018

[fsn370442-bib-0018] Klaunig, J. E. , L. M. Kamendulis , and B. A. Hocevar . 2010. “Oxidative Stress and Oxidative Damage in Carcinogenesis.” Toxicologic Pathology 38, no. 1: 96–109. 10.1177/0192623309356453. 20019356

[fsn370442-bib-0019] Korbecki, J. , I. Baranowska‐Bosiacka , I. Gutowska , and D. Chlubek . 2013. “The Effect of Reactive Oxygen Species on the Synthesis of Prostanoids From Arachidonic Acid.” Journal of Physiology and Pharmacology 64, no. 4: 409–421. 24101387

[fsn370442-bib-0020] Lemke, J. , S. von Karstedt , J. Zinngrebe , and H. Walczak . 2014. “Getting TRAIL Back on Track for Cancer Therapy.” Cell Death and Differentiation 21: 1350–1364. 24948009 10.1038/cdd.2014.81 PMC4131183

[fsn370442-bib-0021] Liang, Y. , L. He , M. Zhang , et al. 2020. “Preserved Egg Digests Promote the Apoptosis of HT29 and HepG2 Cells.” Food Bioscience 36: 100666.

[fsn370442-bib-0022] Matsuzawa, A. , and H. Ichijo . 2008. “Redox Control of Cell Fate by MAP Kinase: Physiological Roles of ASK1‐MAP Kinase Pathway in Stress Signaling.” Biochimica et Biophysica Acta 1780, no. 11: 1325–1336. 10.1016/j.bbagen.2007.12.011. 18206122

[fsn370442-bib-0023] Micucci, C. , G. Matacchione , D. Valli , S. Orciari , and A. Catalano . 2015. “HIF2α Is Involved in the Expansion of CXCR4‐Positive Cancer Stem‐Like Cells in Renal Cell Carcinoma.” British Journal of Cancer 113, no. 8: 1178–1185. 10.1038/bjc.2015.338. 26439684 PMC4647880

[fsn370442-bib-0024] Min, D. Y. , E. Jung , S. S. Ahn , Y. H. Lee , Y. Lim , and S. Y. Shin . 2020. “Chrysoeriol Prevents TNFα‐Induced CYP19 Gene Expression via EGR‐1 Downregulation in MCF7 Breast Cancer Cells.” International Journal of Molecular Sciences 21, no. 20: 7523. 10.3390/ijms21207523. 33053908 PMC7588959

[fsn370442-bib-0025] Murata, M. , R. Thanan , N. Ma , and S. Kawanishi . 2012. “Role of Nitrative and Oxidative DNA Damage in Inflammation‐Related Carcinogenesis.” Journal of Biomedicine and Biotechnology 2012: 1–11. 10.1155/2012/623019. 22363173 PMC3272848

[fsn370442-bib-0026] Nalli, A. D. , L. E. Brown , C. L. Thomas , and T. J. Sayers . 2018. “Sensitization of Renal Carcinoma Cells to TRAIL‐Induced Apoptosis by Rocaglamide and Analogs.” Scientific Reports 8: 17519. 30504817 10.1038/s41598-018-35908-0 PMC6269514

[fsn370442-bib-0027] Obaidi, I. , H. Cassidy , V. I. Gaspar , et al. 2020. “Curcumin Sensitizes Kidney Cancer Cells to TRAIL‐Induced Apoptosis via ROS Mediated Activation of JNK‐CHOP Pathway and Upregulation of DR4.” Biology (Basel) 9, no. 5: 92. 10.3390/biology9050092. 32370057 PMC7284747

[fsn370442-bib-0028] Padala, S. A. , A. Barsouk , K. C. Thandra , et al. 2020. “Epidemiology of Renal Cell Carcinoma.” World Journal of Oncology 11, no. 3: 79–87. 10.14740/WJON1279. 32494314 PMC7239575

[fsn370442-bib-0029] Qiao, J. , Z. Liu , C. Dong , et al. 2019. “Targeting Tumors With IL‐10 Prevents Dendritic Cell‐Mediated CD8+ T Cell Apoptosis.” Cancer Cell 35: 901–915. 31185213 10.1016/j.ccell.2019.05.005

[fsn370442-bib-0030] Tang, T. T. 2002. “The Forkhead Transcription Factor AFX Activates Apoptosis by Induction of the BCL‐6 Transcriptional Repressor.” Journal of Biological Chemistry 277, no. 16: 14255–14265. 10.1074/jbc.M110901200. 11777915

[fsn370442-bib-0031] Voutsadakis, I. A. 2017. “Proteasome Expression and Activity in Cancer and Cancer Stem Cells.” Tumor Biology 39: 10–45. 10.1177/1010428317692248. 28345458

[fsn370442-bib-0032] Wang, Y. , S. N. Sun , Q. Liu , et al. 2016. “Autocrine Complement Inhibits IL10‐ Dependent T‐Cell‐Mediated Antitumor Immunity to Promote Tumor Progression.” Cancer Discovery 6: 1022–1035. 27297552 10.1158/2159-8290.CD-15-1412 PMC5010476

[fsn370442-bib-0033] Wei, W. , J. He , H. Ruan , and Y. Wang . 2019. “In Vitro and In Vivo Cytotoxic Effects of Chrysoeriol in Human Lung Carcinoma Are Facilitated Through Activation of Autophagy, Sub‐G1/G0 Cell Cycle Arrest, Cell Migration and Invasion Inhibition and Modulation of MAPK/ERK Signalling Pathway.” Journal of BUON 24: 936–942. 31424645

[fsn370442-bib-0034] Woo, S. M. , K. J. Min , and T. K. Kwon . 2020. “Inhibition of Drp1 Sensitizes Cancer Cells to Cisplatin‐Induced Apoptosis Through Transcriptional Inhibition of c‐FLIP Expression.” Molecules 25, no. 24: 5793. 10.3390/MOLECULES25245793. 33302576 PMC7764428

[fsn370442-bib-0035] Zeng, X. , J. Shi , M. Zhao , et al. 2016. “Regioselective Glucuronidation of Diosmetin and Chrysoeriol by the Interplay of Glucuronidation and Transport in UGT1A9‐Overexpressing HeLa Cells.” PLoS One 11: e0166239. 27832172 10.1371/journal.pone.0166239 PMC5104480

[fsn370442-bib-0036] Zhang, S. , Z. Chen , P. Shi , et al. 2021. “Downregulation of Death Receptor 4 Is Tightly Associated With Positive Response of EGFR Mutant Lung Cancer to EGFR‐Targeted Therapy and Improved Prognosis.” Theranostics 11, no. 8: 3964–3980. 10.7150/thno.54824. 33664875 PMC7914351

[fsn370442-bib-0037] Zhao, J. , Q. He , Z. Gong , S. Chen , and L. Cui . 2016. “Niclosamide Suppresses Renal Cell Carcinoma by Inhibiting Wnt/β‐Catenin and Inducing Mitochondrial Dysfunctions.” Springerplus 5, no. 1: 1436. 10.1186/s40064-016-3153-x. 27652012 PMC5005241

